# A New Species of *Muscicapa* Flycatcher from Sulawesi, Indonesia

**DOI:** 10.1371/journal.pone.0112657

**Published:** 2014-11-24

**Authors:** J. Berton C. Harris, Pamela C. Rasmussen, Ding Li Yong, Dewi M. Prawiradilaga, Dadang Dwi Putra, Philip D. Round, Frank E. Rheindt

**Affiliations:** 1 Environment Institute and School of Earth and Environmental Sciences, University of Adelaide, Adelaide, South Australia, Australia; 2 Woodrow Wilson School of Public and International Affairs, Princeton University, Princeton, New Jersey, United States of America; 3 Michigan State University Museum and Department of Integrative Biology, East Lansing, Michigan, United States of America; 4 Bird Group, Department of Life Sciences, Natural History Museum at Tring, Tring, Herts, United Kingdom; 5 Fenner School of Environment and Society, Australian National University, Acton, Australian Capital Territory, Australia; 6 South-east Asian Biodiversity Society, Singapore, Republic of Singapore; 7 Division of Zoology, Research Centre for Biology, Lembaga Ilmu Pengetahuan Indonesia, Cibinong-Bogor, West Java, Indonesia; 8 Celebes Bird Club, Palu, Central Sulawesi, Indonesia; 9 Department of Biology, Faculty of Science, Mahidol University, Bangkok, Thailand; 10 Department of Biological Sciences, National University of Singapore, Singapore, Republic of Singapore; BiK-F Biodiversity and Climate Research Center, Germany

## Abstract

The Indonesian island of Sulawesi, a globally important hotspot of avian endemism, has been relatively poorly studied ornithologically, to the extent that several new bird species from the region have been described to science only recently, and others have been observed and photographed, but never before collected or named to science. One of these is a new species of *Muscicapa* flycatcher that has been observed on several occasions since 1997. We collected two specimens in Central Sulawesi in 2012, and based on a combination of morphological, vocal and genetic characters, we describe the new species herein, more than 15 years after the first observations. The new species is superficially similar to the highly migratory, boreal-breeding Gray-streaked Flycatcher *Muscicapa griseisticta*, which winters in Sulawesi; however, the new species differs strongly from *M. griseisticta* in several morphological characters, song, and mtDNA. Based on mtDNA, the new species is only distantly related to *M. griseisticta*, instead being a member of the *M. dauurica* clade. The new species is evidently widely distributed in lowland and submontane forest throughout Sulawesi. This wide distribution coupled with the species' apparent tolerance of disturbed habitats suggests it is not currently threatened with extinction.

## Introduction

The Indonesian island of Sulawesi and its satellite islands are of great biogeographic interest due to the region's complex geography which arose from the collision of several tectonic plates [1],[2]. Stemming from this geological history, Sulawesi is an important center of avian endemism with 42 species (about one-sixth of its resident avifauna) and 14 genera found nowhere else in the world [3],[4]. This level of avifaunal endemism is unmatched globally among comparably sized tropical islands [5].

Sulawesi remains ornithologically one of Earth's least-known areas, as evidenced by the number of recent avian discoveries, especially considering that c. 98% of the world's birds have been described [6]. Recently described species from the Sulawesi region include Cinnabar Hawk-owl *Ninox ios* [7], Sangihe Scops-owl *Otus collari* [8], Togian Hawk-owl *N. burhani* [9], Togian White-eye *Zosterops somadikartai* [10], and two new rails from the Talaud Islands [11],[12]. In addition, several unnamed avian taxa in the region have been observed, but not yet described (e.g. [3],[13]–[16]). The high levels of endemism in Sulawesi combined with alarming habitat loss (10.8% deforestation from 2000–2010; [17]) suggest that many of the island's birds, particularly species dependent on lowland forests, may be threatened with extinction [18], some of them before they are even scientifically described.

**Figure 1:**
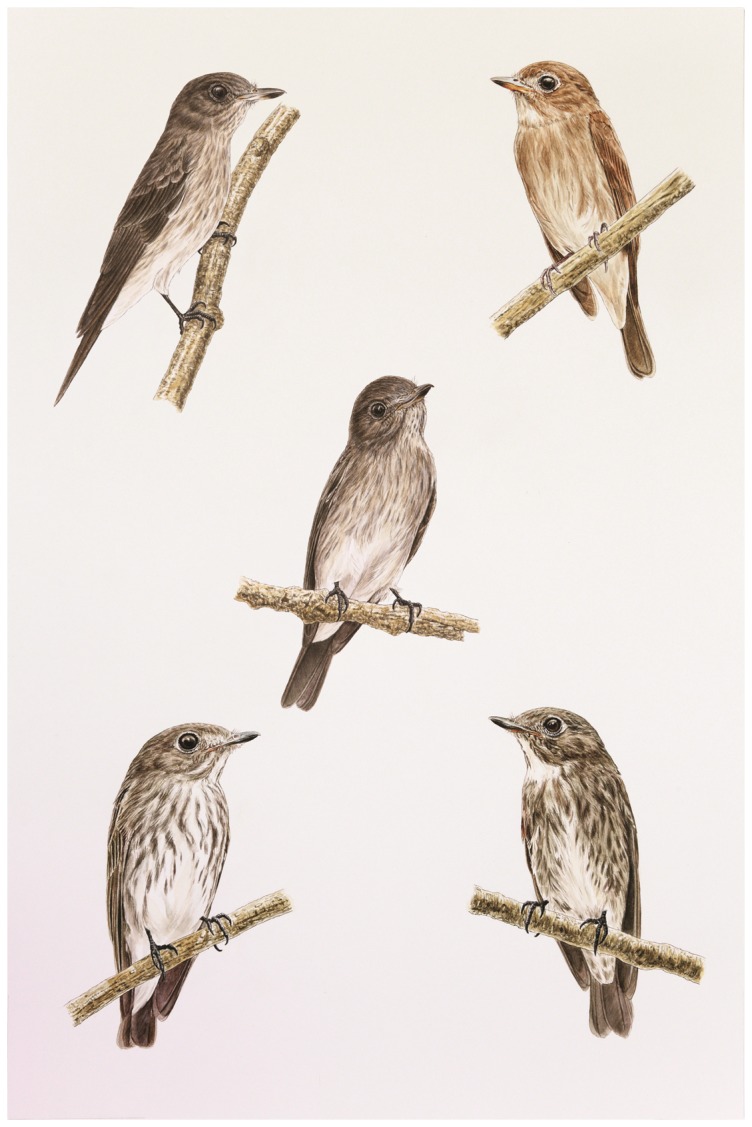
*Muscicapa sodhii* (sp. nov.; Sulawesi Streaked Flycatcher; upper left and center) in comparison to *M. dauurica williamsoni* (Asian Brown [Brown-streaked] Flycatcher; upper right), *M. s. sibirica* (Dark-sided Flycatcher; lower right), and *M. griseisticta* (Gray-streaked Flycatcher; lower left). Original painting by Teo Nam Siang.

**Figure 2:**
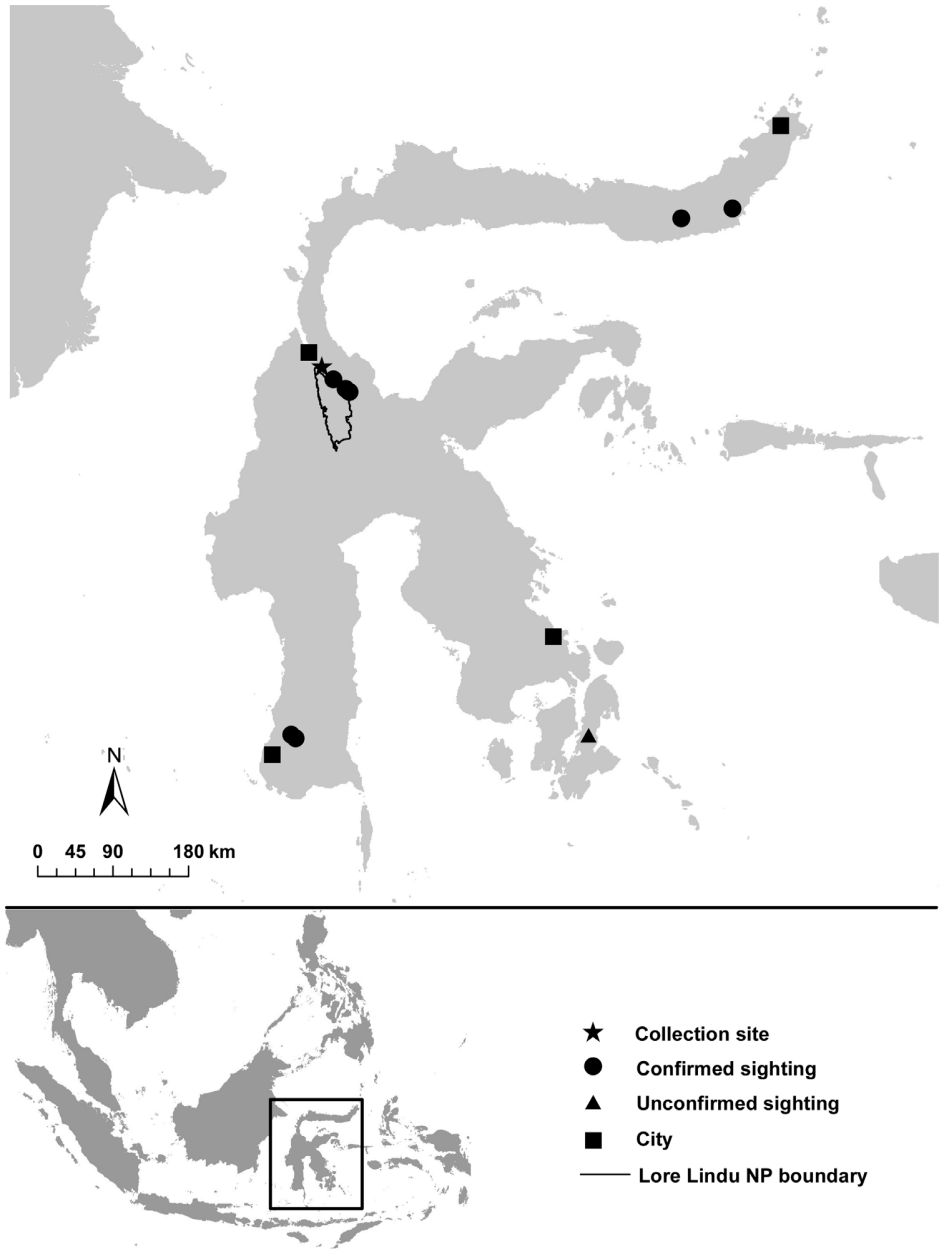
Map of Sulawesi showing records of the new species of *Muscicapa* flycatcher and the type locality.

One of these new birds, a *Muscicapa* flycatcher, has been awaiting formal scientific description for 15 years since it was first documented. While birdwatching in secondary broadleaf forest with some remnant large trees in Lore Lindu National Park, Central Sulawesi, at 1,025 m elevation on 20 Jul 1997, King et al. [19] observed a bird that looked similar to the migratory Gray-streaked Flycatcher *M. griseisticta* (Figure 1). However since it was observed during the boreal summer when migrants were back in their breeding grounds in northern Asia, the record would be highly unusual if it referred to *M. griseisticta*. King et al. [19] described details of the bird, including the fact that it appeared smaller and shorter-winged than *M. griseisticta*, with a distinctively dusky-streaked throat and plain face pattern. King et al. [19] observed what they believed to be the same putatively undescribed species again on 22 Jul 1997 at 650 m in “a very patchy remnant of forest” at the tiny settlement of Baku Bakulu (“km 31 from Palu, on the road leading south-east from Palu through Lore Lindu National Park”, Palolo district near Lore Lindu (−1.1142 S, 119.9917 E). On 23 Jul, a similar bird was observed 500 km to the northeast at 250 m in Bogani Nani Wartabone National Park, North Sulawesi (Figure 2), in “mixed primary and secondary broadleaf evergreen forest” [19]. Subsequent searches among likely taxa in major ornithological collections have failed to find any misidentified specimens that could represent this taxon (N. J. Collar, pers. comm.; P.C.R., this study). Only a few taxa of *Muscicapa* have previously been included in DNA phylogenies [20]. Subsequent to the observations of King et al. [19], visiting birdwatchers have observed the apparently undescribed species at several additional locations and have even documented evidence of breeding (Table 1).

**Table 1:** List of sight records of the new *Muscicapa* species. NP =  National Park.

Locality	Elevation (m)	Region	Year	Month(s)	Observer/Source
*Dated records*					
Pulau Buton	**-**	SE	1996	Jun–Jul	[47]
Kamarora, Lore Lindu NP	900	C	1997	Jul	[19]
Toraut, Bogani Nani Wartabone NP	**-**	C	1997	Jul	[19]
Kamarora, Lore Lindu NP	900	C	1999	Sep	P. Morris; http://www.warbler.phytoconsult.nl/SulaFC.htm
Dongi-Dongi, Lore Lindu NP	1,200	C	2000	Apr–May	[48]
Napu Valley	**-**	C	2000	May	R. Hoff; http://www.surfbirds.com/trip_report.php?id=375
Lore Lindu NP	**-**	C	2004	Aug	[39]
Bantimurung-Bulusaraung NP	150–300	S	2004	Aug–Sep	J. Hornbuckle, I. Merrill; http://jonathanhornbuckle.webs.com/easternindonesia2004.htm
Bantimurung-Bulusaraung NP*	150–300	S	2005	Sep	[49]
Napu Valley	**-**	C	2006	Sep	M. Lagerqvist; http://www.club300.se/Files/TravelReports/Wallacea2006_ML.pdf
Lake Tambing, Lore Lindu NP	1,675	C	2007	Jun	D.L.Y. pers. obs.
Matabulu, Bolaang Mongondow Timur *	<1,000	N	2009	May	I. Hunowu; http://orientalbirdimages.org/search.php?Bird_ID=2770&Bird_Image_ID=36342&p=7
Bantimurung-Bulusaraung NP *	150–300	S	2009	Sep	N. Voaden; http://ibc.lynxeds.com/species/sulawesi-flycatcher-muscicapa-sp-nova
Bantimurung-Bulusaraung NP*	150–300	S	2009	Sep–Oct	[41]
Bantimurung-Bulusaraung NP*	150–300	S	2009	Sep–Oct	[42]
Bantimurung-Bulusaraung NP	150–300	S	2009	October	M. Lindop; http://orientalbirdimages.org/search.php?Bird_ID=2770&Bird_Image_ID=38961&p=1
Near Makassar	**-**	S	2010	Aug–Sep	D. Farrow; http://www.birdquest-tours.com/pdfs/report/INDONESIA%20(SULAWESI)%20REP%2010.pdf
Bantimurung-Bulusaraung NP	150–300	S	2010	Oct	R. Hutchinson; http://www.birdtourasia.com/pdf%20Reports/Birdtour%20Asia%20Sulawesi%20October%202010.pdf
Lore Lindu NP	**-**	C	2011	Jul–Aug	D. Shackelford; http://www.rockjumperbirding.com/wp-content/media/Trip-Report-Sulawesi-Halmahera-birding-trip-report-2011.pdf
Sedoa, Napu Valley*	1,220	S	2011	Sep–Oct	[43]
Lore Lindu NP	**-**	C	2012	Jun	J. Gregory; http://www.worldbirders.com/index.asp?PageId=212&PageName=Sulawesi-2012
Lake Tambing, Lore Lindu NP	1,675	C	2012	Jul–Aug	H. Hendriks; http://www.birdtours.co.uk/tripreports/indonesia/Sulawesi-4/Sulawesi-july-2012.htm
Bantimurung-Bulusaraung NP	150–300	S	2012	Sep	D. Farrow; http://www.birdquest-tours.com/pdfs/report/INDONESIA%20-SULAWESI-%20REP%2012-ebook.pdf
Bantimurung-Bulusaraung NP	150–300	S	2012	Oct	H. Jacob; http://www.surfbirds.com/trip_report.php?id=2263
Bantimurung-Bulusaraung NP	150–300	S	2013	Sep	R. Hutchinson; http://www.birdtourasia.com/pdf%20Reports/Birdtour%20Asia%20Sulawesi%20and%20Halmahera%202013.pdf
*Undated records*					
Upper Palu Valley	**-**	C	-	May	[3]
Badeaha, Napu Valley	1,370	C	-	-	D.D.P. pers. obs.
Lake Tambing, Lore Lindu NP	1,675	C	-	-	D.D.P. pers. obs.

*Evidence of breeding observed.

As part of field work to study undescribed taxa in Central Sulawesi, we searched for the undescribed *Muscicapa* flycatcher in and around Lore Lindu National Park at Danau Tambing, the Anaso track, Badaeha, and Baku Bakulu in Jul 2011 and Jul 2012. In 2011 we focused on Badaeha because most recent Central Sulawesi sightings came from that area (D.D.P. pers. obs.), but we were unable to find the flycatcher there or at any other site in the national park. Eventually we located an individual of the species at Baku Bakulu on 27 Jul 2011, but we were unable to collect a specimen. In 2012 we returned to Baku Bakulu (which is just outside Lore Lindu National Park) where we made comprehensive but unsuccessful attempts to capture the species with canopy mist nets and ground nets. The tendency of the *Muscicapa* flycatcher to forage in the forest mid-levels and subcanopy prevented it from being readily captured. In fact, we had been camping and netting birds intensively in the area for a full week in 2012 before we had our first sighting of the undescribed flycatcher on 21 Jun, at which point we set canopy mist nets where the bird was sighted. On the same day we obtained sound recordings at Baku Bakulu of an individual that was seen clearly while singing. On 23 Jun 2012 at about 1330 h, a local bird hunter showed us and then gave us one of the undescribed flycatchers that he had just shot with an air rifle at Baku Bakulu. Two days later (25 Jun 2012), and unsolicited, the same hunter brought in another individual of the undescribed flycatcher at 1430 h at Baku Bakulu. We prepared these as skin specimens with associated partial skeletons, tissue samples preserved in ethanol, and stomach contents saved in ethanol.

## Materials and Methods

All fieldwork was conducted according to relevant guidelines by the government of the Republic of Indonesia, the USA, and the Republic of Singapore. We thank RISTEK (Indonesia) for issuing the national research permits (for 2011, 0215/SIP/FRP/VI/2011; for 2012, 183/SIP/FRP/SM/VI/2012) and Lore Lindu National Park for the local research permission (SIMAKSI). We obtained approval for our fieldwork from Michigan State University's Institutional Animal Care and Use Committee (IACUC; AUF #05/11-096-00). We obtained the specimens under the relevant Indonesian government collecting permits and in the presence of LIPI ( = Indonesian Institute of Sciences) personnel.

We searched for records of the species from the literature, online audio and photographic databases (xeno-canto, Avian Vocalizations Center, Internet Bird Collection, Oriental Bird Images) and from trip reports from 1996 to present (www.travellingbirder.com, www.birdtours.co.uk, www.cloudbirders.com, www.surfbirds.com, and www.ebird.org).

### Specimens examined

Specimens examined of the new species are listed below as the holotype and paratype. Specimens of congeneric Asian taxa have been examined and measured for comparison as follows (see Acknowledgements for full names of museums): *Muscicapa griseisticta* 47 (38 FMNH, 4 MZB, 5 UMMZ); Dark-sided Flycatcher *Muscicapa s. sibirica* 22 (7 FMNH, 15 USNM); *M. s. gulmergi* 28 (5 BMNH, 14 FMNH, 9 UMMZ); *M. s. cacabata* 33 (22 FMNH, 11 UMMZ); *M. s. rothschildi* 26 (8 AMNH, 10 UMMZ, 8 USNM); Asian Brown Flycatcher *Muscicapa d. dauurica* 14 (14 USNM; the name *Muscicapa dauurica* is available and has priority over *M. latirostris*; [21]); *M. d. umbrosa* 1 (BMNH); *M. d. williamsoni* 15 (11 BMNH, 2 AMNH, 2 YPM); *M. d. siamensis* 11 (3 BMNH, 2 USNM, 6 YPM); Ashy-breasted Flycatcher *Muscicapa randi* 12 [1 BMNH, 2 AMNH, 9 DMNH (4 of these from photographs only)]; Sumba Brown Flycatcher *Muscicapa segregata* 13 (9 AMNH, 3 ZMB, 1 RMNH).

### Morphometric analyses

Measurements taken from each specimen, as condition of the specimen made possible, were (in mm; all measurements made by P.C.R.): culmen length from skull base, culmen length from distalmost feathers; bill width at distal edge of nares; bill depth at distal edge of nares; longest rictal bristle length; approximate extent of pale color at base of lower mandible; length of bill hook; wing length (unflattened); wing length (flattened); primary projection beyond longest secondaries; shortfalls from folded wingtip of primaries 1–10, with P1 the outermost primary; distance between tips of tertial 1 (the shortest) and tertial 2; distance between tips of tertials 2 and 3; distance from feather tip of emargination of P3 (from outside) and P4; shortfall of outer rectrix compared to longest rectrix; tail notch depth; length of tail (with calipers inserted between central rectrices); distance from longest undertail covert to tail tip; tarsus length (to last undivided tarsal scute); and hindclaw length (from edge of last scute). We used a principal components analysis (prcomp function in R) [22] to group taxa based on information from multiple morphological variables. Because of the very small sample size for several taxa, especially the new species, we refrained from statistical testing.

### Plumage analysis

Plumage of all specimens of the above-listed taxa at the above museums was examined in comparison with photographs of the new species, the specimens of which could not be borrowed from the MZB. For the taxa most similar to the new species in plumage, and the other resident tropical and subtropical taxa, plumage scoring was done on a 10-point scale for each characteristic, as possible based on condition of the specimen. Plumage scoring was done on the new species (n = 2), *M. griseisticta* (n = 15), *M. d. umbrosa* (n = 1), *M. d. williamsoni* (n = 9), *M. randi* (n = 3), and *M. segregata* (n = 12). The following plumage characteristics were scored: eyering prominence, from none to prominent; prominence of pale lores, from not differentiated from surrounds to very prominent; degree of crown speckling, from none to prominent; prominence of malar stripe (the dark stripe on the side of the throat, sensu ref. [23]), from not different than surrounds to very prominent; upperparts saturation, from cold gray to rufous-brown; upperparts darkness, from very dark to very pale; throat color, from pure white to all-brown; throat spotting or streaking, from none to heavy; breast color, from pure white to all brown; breast streaking, from none to heavy; central belly color, from pure white to pure brown; vent color, from pure white to pure brown; prominence of vent markings, from none to heavy; rump color, from same as mantle to much more rufous; pale tertial edgings, from none to very prominent; pale edgings of secondary coverts, from none to very prominent. We analyzed these plumage scores with univariate statistics and in a separate principal components analysis.

### Analysis of vocalizations

We recorded the song of one individual of the new species, and compared this to songs of other Asian species of *Muscicapa*, except for the Spotted Flycatcher *Muscicapa striata* and Rusty-tailed Flycatcher *Muscicapa ruficauda*, which have very different songs from each other and their congeners and were therefore omitted (Table 2). We did not obtain any recordings of call notes of the new species, and hence limit our interspecific comparisons to songs. Measurements were taken in Raven Pro 1.3, using the Cool color spectrum to facilitate visualization of maximum power and resonance. Measurements taken for a representative sample for each species were: maximum and minimum note frequencies; frequency of maximum power for a given note; number of consecutive repetitions of a single note; length of note(s); internote interval; intermotif interval; maximum note bandwidth; interstrophe interval; and strophe length. Other aspects of song analyzed were, scored from 0–10: clarity; degree of frequency modulation; harmonic strength; frequency trend within a note; directly counted: number of harmonics; number of elements per motif (phrase); number of motifs per strophe; number of note-types per strophe; or represented by a percentage: degree of similarity of note types within a strophe to the preceding strophe. For vocal analysis univariate statistics and principal components analyses (using a correlation matrix) were conducted using SYSTAT. We used a Kruskal-Wallis One-way Analysis of Variance to test for statistical differences between songs of the new species and each of the other taxa. Although sample sizes of singing individuals represented are very small because most of these species sing infrequently, the number of notes and strophes were sufficient for statistical testing.

**Table 2:** Recordings of songs of Asian *Muscicapa* species analyzed herein.

Species	Cat. No.	Locality	Date	Recordist(s)
new species	AV 17423	Indonesia: Sulawesi; Palolo, Baku Bakulu	21 Jun 2012	P.C.R./J.B.C.H.
new species	AV 17424	Baku Bakulu	21 Jun 2012	P.C.R./J.B.C.H.
new species	AV 17425	Baku Bakulu	21 Jun 2012	P.C.R./J.B.C.H.
new species	AV 17426	Baku Bakulu	21 Jun 2012	P.C.R./J.B.C.H.
new species	AV 17427	Baku Bakulu	21 Jun 2012	P.C.R./J.B.C.H.
new species	AV 17428	Baku Bakulu	21 Jun 2012	P.C.R./J.B.C.H.
*M. griseisticta*	ML 100139	Russian Federation; Primorskiy kray, Chuguyevskyy Rayon, Settlement Chuguyevka	30 May 1986	B. Veprintsev
*M. d. dauurica*	NSA W26730	Russian Federation: Siberia; Irkutsk	10 Jul 1983	L. Svensson
*M. d. dauurica*	—	—		[50]
*M. d.* cf. *siamensis*	XC 49775	Cambodia: Preăh Vihéar Province; Chhep	14 Mar 2002	D. Farrow
*M. randi*	AV 14197	Philippines: Luzon I.; Sierra Madre Mts., Hamut Camp	18 Apr 2011	B. Demeulemeester
*M. randi*	AV 14368	Hamut Camp	18 Apr 2011	B. Demeulemeester
*M. randi*	NSA 66239	Hamut Camp	20 Feb 1996	P. Morris
*M. segregata*	—	Indonesia: Sumba; Langiliuru National Park, 500 m	Sep 2013	J. Eaton
*M. segregata*	NSA 46748	Indonesia: Sumba; Lewa	22 Oct 1992	A. Lewis
*M. s. sibirica*	—	—	—	[50]
*M. muttui*	AV 6672	Sri Lanka: Central Province; Kandy, Udawattakele Sanctuary	27 Feb 2004	D. Warakagoda
*M. ferruginea*	ML13910	Taiwan: Nantou	30 Apr 1967	S. R. Severinghaus
*M. ferruginea*	XC 34200	Taiwan: Dasyueshan	25 Apr 2009	F. R. Lambert
*M. ferruginea*	NSA 34492	Taiwan: Nantou	3 Jun 1985	Liou Yih-Hwa
*M. ferruginea*	NSA 34493	Taiwan: Nantou	8 Jul 1991	Liou Yih-Hwa

### DNA sequencing and analysis

DNA of the two specimens from Sulawesi was extracted from fresh tissue (from pectoral muscle samples taken from the specimens; Table 3) using a DNeasy Blood & Tissue kit from Qiagen. DNA of a *M. dauurica siamensis* individual was extracted using the same lab methodology from a single rectrix feather supplied by P.D.R. from a bird mist-netted on 23 May 2011 at a known breeding site in Doi Chiang Dao, Chiang Mai Province, northern Thailand and subsequently released after being measured, photographed, and banded (Table 3). Muscle tissue of a further 13 specimens of various boreal *Muscicapa* species was loaned from the Burke Museum of Natural History and Culture (University of Washington, Seattle; Table 3) and their DNA was extracted using the same lab procedures. COI primers used were those of Kerr et al. [24]. Sequencing procedures followed those in Rheindt et al. [25] and/or Kerr et al. [24]. Sequences have been uploaded to Genbank under the accession numbers KM924371 to KM924385. Sequences were easily aligned by eye as they did not contain any indels. Our COI sequence alignment was 708 bp long and spanned 15 *Muscicapa* individuals belonging to six species. All phylogenetic analysis was carried out in Mega5 [26] with 1000 bootstrap replicates: maximum-parsimony analysis was conducted using the tree-bisection-reconnection method, while Neighbor-joining analysis was implemented with a pairwise deletion treatment for gaps and a raw p-divergence model; thirdly, maximum-likelihood analysis was conducted using a General Time Reversible model with invariant sites as this was the most likely model rendered by jModeltest [27] that is implemented in Mega5. Raw p-divergences were computed with Mega5 using pairwise deletion for gaps.

**Table 3:** Specimens for DNA analysis.

Taxon	Country or island of collection	Housing institution and/or collector	Specimen number or voucher number	Tissue type
*M. d. dauurica*	Russia	BMNHC	83274	muscle
*M. d. dauurica*	Russia	BMNHC	83255	muscle
*M. d. dauurica*	Russia	BMNHC	83363	muscle
*M. d. dauurica*	Russia	BMNHC	72147	muscle
*M. d. dauurica*	Russia	BMNHC	80890	muscle
*M. d. siamensis*	Thailand	Philip D. Round	n/a	feather
new species	Sulawesi	MZB	A6 6159	muscle
new species	Sulawesi	MZB	A6 6160	muscle
*M. s. sibirica*	Mongolia	BMNHC	59998	muscle
*M. s. sibirica*	Russia	BMNHC	83285	muscle
*M. s. sibirica*	Russia	BMNHC	83370	muscle
*M. adusta*	South Africa	BMNHC	67144	muscle
*M. griseisticta*	Russia	BMNHC	58404	muscle
*M. griseisticta*	Russia	BMNHC	44144	muscle
*M. griseisticta*	Russia	BMNHC	44071	muscle

Abbreviations: BMNHC – Burke Museum of Natural History and Culture (University of Washington, Seattle, USA); MZB – Museum Zoologicum Bogoriense (Cibinong, West Java, Indonesia).

### Nomenclatural acts

The electronic edition of this article conforms to the requirements of the amended International Code of Zoological Nomenclature [28], and hence the new name contained herein is available under that Code from the electronic edition of this article. This published work and the nomenclatural act it contains have been registered in ZooBank, the online registration system for the ICZN. The ZooBank LSID (Life Science Identifier) can be resolved and the associated information viewed through any standard web browser by appending the LSID to the prefix “http://zoobank.org/”. The LSID for this publication is: urn:lsid:zoobank.org:pub: 19FE6BDD-5F9F-4E16-BCAA-6EADF959DDEB. The electronic edition of this work was published in a journal with an ISSN, and has been archived and is available from the following digital repositories: PubMed Central and LOCKSS.

## Results

### Morphology

#### Morphological and plumage differences

In comparisons of the new species and 11 other *Muscicapa* taxa we found that the new species has a unique combination of morphological characteristics (also see *Diagnosis* below). The new species has (1) shorter wings and wingtip extension than all taxa except *umbrosa*; (2) a more strongly hooked bill than all taxa except for *M. randi* and *M. segregata*; (3) a shorter tail than *M. randi*, *M. segregata*, *M. s. sibirica*, *M. griseisticta*, and *M. d. siamensis*; (4) shorter undertail coverts than *M. d. siamensis*, *M. griseisticta*, and all *M. sibirica* taxa; (5) a longer bill than *M. griseisticta*, *M. d. umbrosa*, and all *M. sibirica* taxa; (6) a less extensive pale area on the lower mandible than *M. randi*, *M. segregata*, *M. d. siamensis*, and *M. d. williamsoni*; (7) a narrower bill than *M. segregata* and *M. d. dauurica*, but a wider bill than all *M. sibirica* taxa except the nominate; (8) a shorter tarsus than *M. segregata* (Table 4).

**Table 4:** Morphometric measurements presented as mean ±SD with sample size (*n*) in parentheses.

Variable	*M. griseisticta*	new species	*M. s. sibirica*	*M. s. gulmergi*	*M. s. cacabata*	*M. s. rothschildi*	*M. dauurica*	*M. d. umbrosa*	*M. d. williamsoni*	*M. d. siamensis*	*M. randi*	*M. segregata*
Culmen from skull	14.0±0.6 (*n* = 36)	15.3±0.6 (*n* = 2)	13.9±0.6 (*n* = 16)	11.9±0.7 (*n* = 23)	11.9±0.7 (*n* = 18)	12.1±0.7 (*n* = 18)	15.0±0.5 (*n* = 13)	13.3 (*n* = 1)	15.5±0.9 (*n* = 12)	14.8±0.4 (*n* = 10)	14.7±0.5 (*n* = 3)	16.3±0.5 (*n* = 13)
Culmen from feathers	9.5±0.6 (*n* = 36)	10.7±0.5 (*n* = 2)	8.1±0.5 (*n* = 16)	7.5±0.4 (*n* = 23)	7.4±0.6 (*n* = 18)	7.5±0.7 (*n* = 17)	9.3±0.6 (*n* = 13)	9.6 (*n* = 1)	10.7±1.0 (*n* = 12)	10.2±0.5 (*n* = 10)	11.0±0.7 (*n* = 3)	11.8±0.3 (*n* = 13)
Bill width from nares	4.3±0.4 (*n* = 40)	4.4±0.1 (*n* = 2)	4.4±0.2 (*n* = 16)	3.6±0.4 (*n* = 23)	3.6±0.3 (*n* = 19)	3.7±0.4 (*n* = 20)	5.0±0.3 (*n* = 14)	4.7 (*n* = 1)	5.0±0.4 (*n* = 15)	4.8±0.7 (*n* = 8)	4.5±0.1 (*n* = 3)	5.2±0.3 (*n* = 13)
Bill depth from nares	3.7±0.2 (*n* = 29)	3.8±0.1 (*n* = 2)	3.0±0.2 (*n* = 16)	2.6±0.3 (*n* = 23)	2.5±0.2 (*n* = 19)	2.6±0.2 (*n* = 20)	3.3±0.4 (*n* = 14)	3.1 (*n* = 1)	3.5±0.2 (*n* = 14)	3.6±0.4 (*n* = 8)	3.6±0.4 (*n* = 3)	3.9±0.1 (*n* = 12)
Longest rictal bristle	5.5±1 (*n* = 31)	7.7±1.8 (*n* = 2)	6.6±0.6 (*n* = 16)	6.3±0.8 (*n* = 24)	6.4±0.7 (*n* = 18)	6.4±0.5 (*n* = 17)	7.1±0.7 (*n* = 14)	5.8 (*n* = 1)	7.5±0.8 (*n* = 13)	7.9±0.4 (*n* = 4)	9.1±2.0 (*n* = 3)	9.3±1.3 (*n* = 10)
Extent of pale area on lower mandible	3.2±2 (*n* = 31)	4.8±0.6 (*n* = 2)	3.7±1.8 (*n* = 15)	3.5±1.0 (*n* = 24)	3.2±0.9 (*n* = 19)	2.8±0.7 (*n* = 15)	5.5±1.1 (*n* = 14)	4.9 (*n* = 1)	6.9±1.5 (*n* = 15)	8.4±1.7 (*n* = 8)	10.5±0.8 (*n* = 3)	7.7±1.2 (*n* = 12)
Bill hook	1.0±0.1 (*n* = 34)	1.4±0.1 (*n* = 2)	0.9±0.1 (*n* = 16)	0.9±0.2 (*n* = 21)	0.9±0.1 (*n* = 17)	0.9±0.1 (*n* = 14)	1.0±0.1 (*n* = 13)	1.2 (*n* = 1)	0.9±0.1 (*n* = 12)	1.1±0.1 (*n* = 5)	1.2±0.2 (*n* = 3)	1.2±0.2 (*n* = 9)
Wing unflattened	84.8±3.1 (*n* = 39)	62.3±1.1 (*n* = 2)	79.8±3.2 (*n* = 16)	72.9±1.6 (*n* = 27)	73.8±1.7 (*n* = 19)	73.5±2.0 (*n* = 20)	69.9±1.9 (*n* = 14)	59.5 (*n* = 1)	70.6±3.2 (*n* = 15)	69.4±1.9 (*n* = 8)	65.7±1.8 (*n* = 3)	66.8±1.5 (*n* = 13)
Wing flattened	85.1±3.1 (*n* = 39)	63±1.4 (*n* = 2)	80.3±3.1 (*n* = 16)	73.1±1.6 (*n* = 27)	74.1±1.7 (*n* = 19)	73.8±2.2 (*n* = 20)	70.4±2.1 (*n* = 14)	60.0 (*n* = 1)	70.9±3.0 (*n* = 15)	69.8±1.7 (*n* = 11)	66.3±1.5 (*n* = 3)	67.1±1.6 (*n* = 13)
Wingtip extension	30.7±3.2 (*n* = 39)	10.4±1.1 (*n* = 2)	26.4±2.8 (*n* = 16)	21.2±1.7 (*n* = 27)	21.4±1.9 (*n* = 19)	21.8±1.8 (*n* = 18)	19.9±1.6 (*n* = 14)	10.3 (*n* = 1)	19.6±4.1 (*n* = 15)	17.8±1.7 (*n* = 11)	14.6±2.2 (*n* = 3)	13.5±0.8 (*n* = 13)
Primary 1	54.2±2.2 (*n* = 39)	33.5±1 (*n* = 2)	50±2.2 (*n* = 16)	42.4±2.1 (*n* = 25)	42.8±1.6 (*n* = 18)	43.4±2.1 (*n* = 20)	38.0±1.7 (*n* = 14)	28.6 (*n* = 1)	38.1±2.0 (*n* = 14)	37.8±1.6 (*n* = 8)	33.8±0.4 (*n* = 3)	32.3±2.3 (*n* = 11)
Primary 2	2.6±0.8 (*n* = 39)	5.8±1.1 (*n* = 2)	3.4±0.7 (*n* = 15)	4.2±1.0 (*n* = 25)	4.6±0.9 (*n* = 18)	4.2±1.1 (*n* = 20)	5.8±1.0 (*n* = 13)	6.7 (*n* = 1)	5.4±0.9 (*n* = 14)	5.7±0.9 (*n* = 8)	7.9±0.8 (*n* = 3)	6.9±0.7 (*n* = 11)
Primary 3	0 (*n* = 39)	1.7 (*n* = 1)	0 (*n* = 16)	0.1±0.3 (*n* = 25)	0 (*n* = 18)	0.03±0.1 (*n* = 20)	0.1±0.3 (*n* = 14)	1 (*n* = 1)	0.1±0.3 (*n* = 14)	0 (*n* = 8)	1.4±0.4 (*n* = 3)	1.3±0.5 (*n* = 11)
Primary 4	2.0±0.5 (*n* = 39)	0 (*n* = 1)	1.5±0.5 (*n* = 16)	0.5±0.7 (*n* = 25)	0.5±0.5 (*n* = 18)	0.7±1.2 (*n* = 19)	0.04±0.1 (*n* = 13)	0 (*n* = 1)	0.1±0.2 (*n* = 14)	0.1±0.4 (*n* = 8)	0.2±0.3 (*n* = 3)	0 (*n* = 11)
Primary 5	8.0±1 (*n* = 26)	0 (*n* = 2)	6.6±0.9 (*n* = 16)	4.0±1.2 (*n* = 25)	3.8±0.8 (*n* = 17)	4.2±0.6 (*n* = 16)	2.9±1.8 (*n* = 13)	1 (*n* = 1)	2.3±0.9 (*n* = 14)	2.5±0.5 (*n* = 7)	0.8±0.7 (*n* = 3)	0.6±0.5 (*n* = 10)
Primary 6	14.8±1.2 (*n* = 38)	2.7±0.5 (*n* = 2)	13.5±1.4 (*n* = 16)	10.2±0.8 (*n* = 25)	10.5±0.8 (*n* = 18)	10.9±1.3 (*n* = 17)	8.6±1.7 (*n* = 12)	3.6 (*n* = 1)	7.6±1.6 (*n* = 13)	7.9±1.2 (*n* = 7)	3.8±1.3 (*n* = 3)	3.2±0.7 (*n* = 10)
Primary 7	19.6±1.2 (*n* = 39)	6.8 (*n* = 1)	18±1.4 (*n* = 16)	14.2±0.9 (*n* = 24)	14.6±0.8 (*n* = 17)	14.9±1.0 (*n* = 17)	12.6±1.2 (*n* = 12)	7.3 (*n* = 1)	11.7±2.0 (*n* = 14)	12.2±1.2 (*n* = 7)	8.5±1.8 (*n* = 3)	7.3±0.7 (*n* = 10)
Primary 8	23.3±1.5 (*n* = 39)	9.0±0.1 (*n* = 2)	21.3±1.4 (*n* = 16)	17.0±1.2 (*n* = 24)	17.4±0.9 (*n* = 17)	17.8±1.1 (*n* = 15)	15.4±1.2 (*n* = 12)	8.9 (*n* = 1)	14.7±1.9 (*n* = 13)	14.6±1.4 (*n* = 8)	11.2±1.5 (*n* = 3)	10.0±1.1 (*n* = 10)
Primary 9	26.7±1.5 (*n* = 38)	10.5±0.1 (*n* = 2)	24.1±1.4 (*n* = 16)	19.2±1.1 (*n* = 22)	19.5±0.9 (*n* = 16)	20.0±1 (*n* = 16)	17.4±1.1 (*n* = 12)	*N*A	17.1±2.1 (*n* = 13)	16.7±1.0 (*n* = 8)	13.0±1.4 (*n* = 3)	11.9±1.2 (*n* = 10)
Primary 10	29.8±1.7 (*n* = 38)	12.6±0 (*n* = 2)	27.0±1.5 (*n* = 16)	21.5±1.2 (*n* = 21)	21.9±0.8 (*n* = 17)	22.3±1.2 (*n* = 16)	19.7±1.3 (*n* = 12)	*N*A	19.7±2.1 (*n* = 13)	18.8±1.0 (*n* = 8)	14.3±1.6 (*n* = 3)	13.6±1.4 (*n* = 10)
Tertial 1 to 2	7.7±1.1 (*n* = 41)	5.4±0.9 (*n* = 2)	8.3±0.9 (*n* = 16)	7.2±2.3 (*n* = 21)	7.4±1.1 (*n* = 17)	8.2±0.9 (*n* = 18)	8.7±1.5 (*n* = 13)	5.3 (*n* = 1)	6.5±1.5 (*n* = 12)	7.0±1.1 (*n* = 7)	6.5±2.2 (*n* = 2)	9.4±0.7 (*n* = 11)
Tertial 2 to 3	8.2±1.1 (*n* = 41)	10.9±1.6 (*n* = 2)	6.6±1.1 (*n* = 16)	6.1±1.7 (*n* = 22)	5.2±1.4 (*n* = 16)	5.4±0.9 (*n* = 18)	6.6±0.8 (*n* = 13)	5.9 (*n* = 1)	8.0±1.5 (*n* = 12)	7.2±1.8 (*n* = 7)	7.1±1.1 (*n* = 2)	5.3±1.0 (*n* = 11)
Emargination 3	22.1±2.8 (*n* = 37)	23.0 (*n* = 1)	22.3±1.5 (*n* = 15)	22.8±1.9 (*n* = 24)	23.3±1.4 (*n* = 19)	23.0±1.8 (*n* = 17)	22.9±1.5 (*n* = 12)	23.6 (*n* = 1)	23.1±1.6 (*n* = 12)	23.0±1.0 (*n* = 7)	24.6±1.5 (*n* = 3)	24.7±2.5 (*n* = 8)
Emargination 4	15.9±1.6 (*n* = 32)	21.6±2.1 (*n* = 2)	15.6±1.8 (*n* = 15)	17±1.8 (*n* = 24)	17.9±1.6 (*n* = 18)	17.8±1.2 (*n* = 16)	17.6±2.0 (*n* = 12)	17.9 (*n* = 1)	17.6±1.7 (*n* = 13)	17.4±1.3 (*n* = 7)	21.1±0.9 (*n* = 3)	20.9±2.3 (*n* = 9)
Outer rectrix to longest rectrix	3.8±1.2 (*n* = 39)	2.5±0.6 (*n* = 2)	0.7±0.7 (*n* = 16)	4.0±1.3 (*n* = 27)	3.9±1.2 (*n* = 18)	4.6±0.9 (*n* = 19)	2.8±1.1 (*n* = 14)	2.0 (*n* = 1)	1.8±1.4 (*n* = 14)	2.1±1.1 (*n* = 5)	2.4±0.9 (*n* = 3)	1.2±1.4 (*n* = 12)
Tail graduation (notch)	0.8±0.7 (*n* = 37)	1.5±0.7 (*n* = 2)	5.0±0.8 (*n* = 15)	0.4±0.5 (*n* = 27)	0.5±0.6 (*n* = 18)	0.4±0.6 (*n* = 20)	0.8±0.7 (*n* = 13)	2.5 (*n* = 1)	1.5±0.9 (*n* = 14)	1.6±0.7 (*n* = 6)	1.7±0.6 (*n* = 3)	2.7±1.1 (*n* = 12)
Tail length	47.8±2.7 (*n* = 39)	44.3±0.4 (*n* = 2)	50.2±2.6 (*n* = 16)	44.2±1.9 (*n* = 27)	45.5±1.8 (*n* = 18)	46.1±1.7 (*n* = 19)	46.1±2.1 (*n* = 14)	48.2 (*n* = 1)	46.4±3.1 (*n* = 14)	47.4±1.6 (*n* = 11)	49.9±2.9 (*n* = 3)	49.4±1.9 (*n* = 13)
Undertail coverts to tail tip	14.9±3.3 (*n* = 39)	21.5±0.7 (*n* = 2)	15.3±1.8 (*n* = 16)	16.3±2.0 (*n* = 26)	15.9±1.8 (*n* = 18)	16.1±1.1 (*n* = 19)	18.4±2.9 (*n* = 14)	32.6 (*n* = 1)	19.3±2.0 (*n* = 13)	18.3±2.3 (*n* = 7)	26.0±2.5 (*n* = 2)	25.2±2.4 (*n* = 11)
Tarsus length	13.8±0.7 (*n* = 41)	13±0.6 (*n* = 2)	13.4±0.6 (*n* = 16)	11.9±0.8 (*n* = 28)	12.1±1.0 (*n* = 16)	12.2±0.7 (*n* = 18)	13.7±0.7 (*n* = 14)	11.2 (*n* = 1)	13.4±0.6 (*n* = 15)	13.4±0.7 (*n* = 11)	14.1±1.2 (*n* = 3)	14.6±0.6 (*n* = 13)
Hindclaw length	4.6±0.3 (*n* = 38)	4.4±0.3 (*n* = 2)	4.4±0.3 (*n* = 16)	3.9±0.3 (*n* = 28)	4.0±0.2 (*n* = 17)	3.9±0.3 (*n* = 16)	4.2±0.3 (*n* = 14)	4.6 (*n* = 1)	4.3±0.4 (*n* = 15)	4.2±0.4 (*n* = 7)	4.7±0.5 (*n* = 3)	4.6±0.3 (*n* = 12)

**Figure 3:**
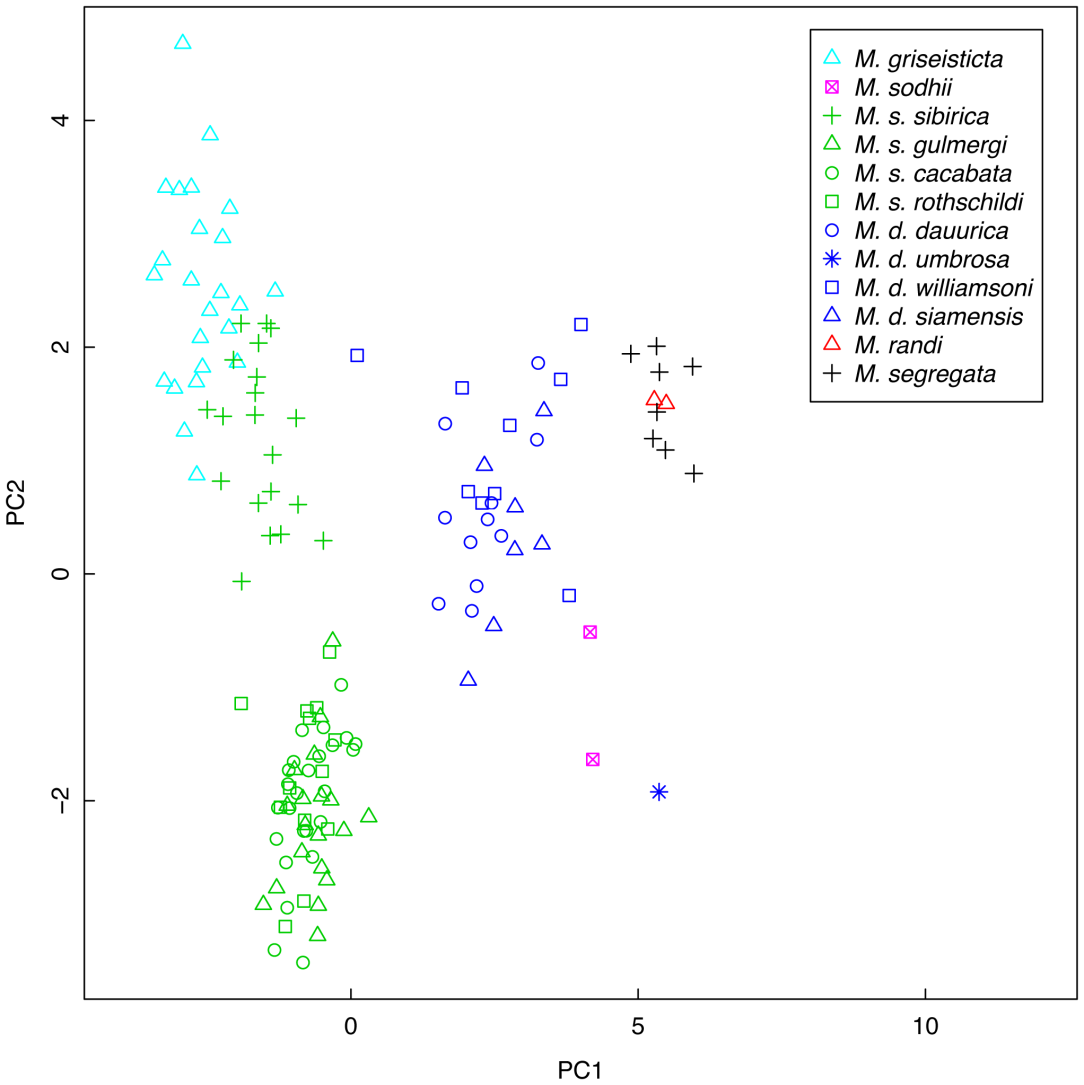
Principal components analysis of morphometric measurements of Asian *Muscicapa* taxa.

When the morphological variables were combined in a principal components analysis, clusters of individuals from each species-level taxon, including the new species, were clearly separated (Figure 3). The PCA suggests that the new species is most similar to the other sedentary taxa and *M. d. dauurica* in structure. On Factor 1, which is a shape axis that contrasts primary 2 shortfall, undertail coverts shortfall, and amount of pale on lower mandible against wing measures (Table 5), the new species and *M. griseisticta* are very far apart, despite their superficial similarity in plumage, primarily due to their very different wing shapes. On Factor 2, which is a size axis, to which only primary 2 length is negatively correlated and undertail coverts shortfall is uncorrelated, the new species is distinct from all *M. segregata*, the lone *M. d. umbrosa*, and *M. randi*, but because the new species is larger than those taxa in some variables and smaller in others, this axis is not readily interpretable, especially given small sample sizes. We also note here that *Muscicapa s. sibirica* is distinct in morphology from *M. s. cacabata* and *M. s. gulmergi* on both axes.

**Table 5:** Component loadings for principal components analysis of morphometric measurements of Asian *Muscicapa* taxa.

Variable	Factor 1	Factor 2
wing flattened	***−0.33***	**0.27**
wingtip extension	***−0.34***	0.24
primary 1	***−0.36***	0.20
primary 2	***0.33***	−0.09
primary 8	***−0.37***	0.18
tail	0.00	**0.31**
undertail coverts to tail tip	***0.34***	0.02
culmen from skull	**0.21**	***0.39***
culmen from feathers	**0.26**	**0.33**
bill width	**0.21**	**0.33**
extent of pale on lower mandible	***0.33***	0.12
hindclaw	0.05	**0.38**
tarsus	0.09	**0.41**
eigenvalues	2.40	1.94
percent total variance explained	44.49	28.86

Variables deemed most important in bold italic, those of somewhat lesser importance in bold only, and those not considered important in regular font.

**Figure 4:**
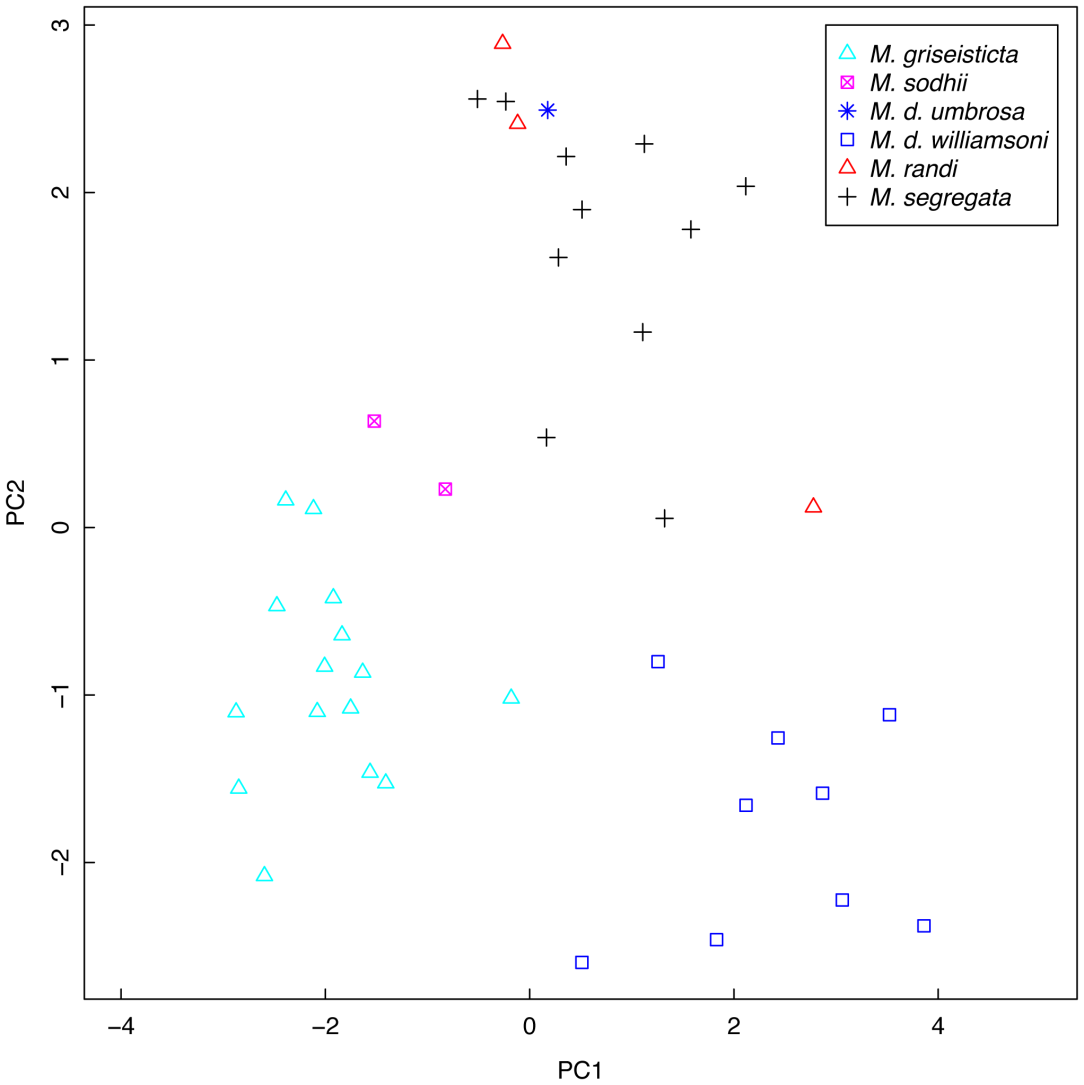
Principal components analysis of plumage scores of Asian *Muscicapa* taxa. Specimens were scored from 1–10 based on 13 plumage traits. See Table 6 for details.

Plumage scoring comparisons with *M. d. umbrosa*, *M. d. williamsoni*, *M. griseisticta*, *M. randi*, and *M. segregata* showed that the new species has (1) stronger crown speckling than all other taxa; (2) heavier breast streaking than *M. d. umbrosa*, *M. randi*, or *M. segregata*; (3) a much weaker malar than *M. griseisticta*; (4) a less contrasting rump than *M. d. williamsoni* or *M. randi*; (5) a less prominent eyering than *M. williamsoni*; (6) weaker spotting on secondary coverts than *M. d. umbrosa* or *M. d. williamsoni*; (7) paler upperparts than *M. d. umbrosa*; and (8) colder brown upperparts than *M. d. williamsoni* (Table 6). Principal components analysis of the plumage scores showed clear separation of the new species, *M. griseisticta*, and *M. d. williamsoni* from each other and the other taxa (Figure 4). On Factor 1, which primarily contrasts upperparts color with degree of marking on crown, malar, and breast (Table 7), the new species falls between the less-marked *M. segregata* and the more strongly marked *M. griseisticta*. On Factor 2, which primarily contrasts ground color of the breast with prominence of eyering, malar, and breast streaking, the new species is fairly similar to all taxa except *M. d. williamsoni*, which has a much warmer brown breast.

**Table 6:** Plumage scores for six Asian *Muscicapa* taxa presented as mean ± SD with sample size (*n*) in parentheses.

Variable	*M. griseisticta* (*n* = 15)	new species (*n* = 2)	*M. d. umbrosa* (*n* = 1)	*M. d. williamsoni* (*n* = 9)	*M. randi* (*n* = 3)	*M. segregata* (*n* = 12)
eyering (1 = none, 10 = prominent)	4.3±1.2	3	4	5.7±1.3	2±1.7	3.1±0.9
crown speckling (1 = none, 10 = prominent)	3.7±1.3	6	1	1.8±1.2	3.3±1.2	2.4±1.2
breast color (1 = pure white, 10 = all brown)	4.9±0.6	6	9	5.7±1.1	8±1	8.3±0.97
breast streaking (1 = none, 10 = heavy)	8±0.76	7	2	6±1.4	2±1	2.5±1.4
vent color (1 = pure white, 10 = pure brown)	1.1±0.26	1	1	1.1±0.3	1	1.2±0.39
vent marking (1 = none, 10 = heavy)	1.1±0.26	1	1	1	1	1
malar (1 = none, 10 = strong)	7.5±0.92	1	1	3.2±2.2	1.3 ±0.58	1.4±0.67
rump (1 = as mantle, 10 = much more rufous)	1	1	3	5.4±1.2	3±1	1.3±0.65
tertials (1 = plain, 10 = broad strong pale margins)	4±1.9	6±2.8	1	7.6±1.7	5.7±4	4.3±2.1
upperparts darkness (1 = very dark, 10 = very pale)	5±0.38	5.5±0.7	3	5.4±0.7	5.3 ±0.58	5.3±0.98
upperparts hue (1 = cold, 10 = rufous)	3.1±0.52	2.5±0.7	2	6.8±1.1	3.3±0.58	4.5±1
secondary coverts spotting (1 = none, 10 = strong)	3.3±1.3	2	8	6.4±2.4	4.7±4.6	4.4±2.5
lores (1 = no pale, 10 = broad pale)	3.8±1.4	6±1.4	3	5.8±1.1	5.3±2.3	4.5±1.7

**Table 7:** Component loadings for principal components analysis of plumage scores for six Asian *Muscicapa* taxa.

Variable	Factor 1	Factor 2	Factor 3	Factor 4
eyering	0.07	***−0.47***	0.05	−**0.15**
crown speckling	***−0.33***	−0.02	***−0.31***	***0.31***
breast color	**0.20**	***0.49***	0.08	−0.02
breast streaking	**−0.25**	***−0.45***	0.01	−0.04
vent color	0.06	−0.04	***0.61***	***0.40***
vent marking	−0.11	−0.12	***0.53***	***0.37***
malar	***−0.34***	***−0.37***	0.01	**−0.15**
rump	***0.37***	**−0.24**	0.01	**−0.24**
tertials	***0.33***	**−0.25**	−0.19	**0.20**
upperparts darkness	**0.18**	−0.07	***−0.39***	***0.51***
upperparts hue	***0.42***	**−0.17**	−0.05	0.09
secondary coverts spotting	***0.36***	−0.08	**0.21**	***−0.31***
lores	**0.25**	**−0.15**	−0.07	***0.33***
eigenvalues	1.90	1.68	1.25	1.18
percent total variance explained	27.84	21.70	11.94	10.70

Variables deemed most important in bold italic, those of somewhat lesser importance in bold only, and those not considered important in regular font. See Table 6 for more details.

### Song

**Figure 5:**
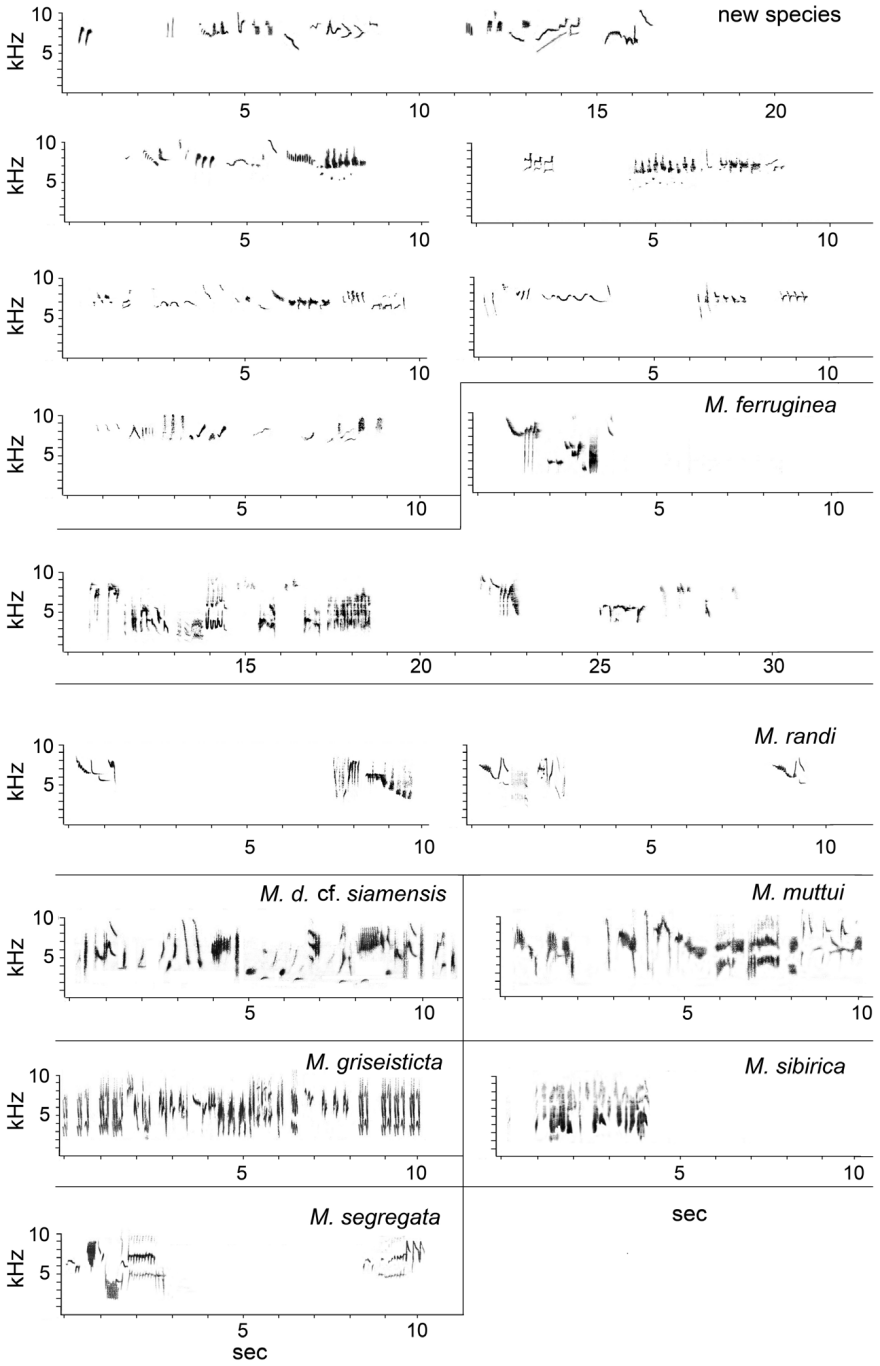
Sonagrams of the new species and related Asian taxa. Localities, recordists, and catalog number (if any): new species, Baku Bakulu, Sulawesi, P.C.R./J.B.C.H., AV17423–17428; successive strophes from the same individual; *M. ferruginea*, Nantou, Taiwan, S. Severinghaus, ML 13910; *M. randi*, Hamut, Luzon, B. Demeulemeester, AV 14197, 14368; *M. d.* cf. *siamensis*, Cambodia, D. Farrow, XC 49755; *M. muttui*, Kandy, Sri Lanka, D. Warakagoda, AV 6672; *M. griseisticta*, Russia, B. Veprintsev, ML 100139; *M. s. sibirica*, H. Ueda; *M. segregata*, Sumba, J. Eaton.

The song of the new species (http://avocet.zoology.msu.edu/recordings/17424 through 17428) is similar to those of some of its congeners, but differs in being slightly to much higher-pitched, and mostly within a narrower bandwidth (c.6–10 kHz) than other *Muscicapa* species (Figure 5; Table 8). It consists of thin, very high-pitched whistles, chirps, twitters, glissandos, buzzy notes and trills (among other note types), of highly varied and often complex form, often highly modulated, and with little repetition, although individual elements and motifs are often repeated a few times in quick succession, or a motif may be given again several seconds later within a strophe that otherwise differs strongly. The song can further be described as a rather deliberately-paced apparently random amalgam of different note types in a strophe that lasts from less than a second to several seconds, often separated from other strophes by several seconds but also often with such short inter-strophe intervals as to make definition of strophe length unclear.

**Table 8:** Measurements of songs of the new species of *Muscicapa* and closely related Asian congeners.

Variable	new species(*n* = 1 individual)	*M. griseisticta* (*n* = 1 individual; Russia)	*M. d. dauurica* (*n* = 1 individual; Japan)	*M. d.* cf. *siamensis* (*n* = 1 individual; Cambodia)	*M. randi* (*n* = 2–3 individuals; Luzon)	*M. segregata* (*n = *2 individuals; Sumba)	*M. s. sibirica* (*n* = 1 individual; Japan)	*M. muttui* (*n* = 1–2 individuals; Sri Lanka)	*M. ferruginea* (*n* = 3–4 individuals; Taiwan)
Max note f (kHz)	8090.8±877.5 (5995–10183; 76)	4912.8±1601.7^3^ (2138–7736; 27)	5717.4±1681.3^3^ (2315–8725; 37)	7130.0±1187.0^3^ (3849–8954; 46)	6696.4±1186.0^3^ (3469–8603; 83)	6125.2±1898.5^3^ (2257–8694; 43)	4437.3±670.2^3^ (3440–5447; 21)	7422.6±1204.5^1^ (3989–8973; 23)	6382.7±1795.8^3^ (3214–9199; 26)
Min note f (kHz)	7221.1±598.1 (5745–9058; 76)	3117.6±877.4^3^ (1887–5472; 27)	4835.6±1978.7^3^ (2026–8353; 37)	4401.0±1230.0^3^ (2176–7029; 46)	5118.3±1288.9^3^ (2891–8019; 83)	5151.8±1784.1^3^ (1715–8226; 43)	3176.8±361.9^3^ (2711–3774; 21)	6195.4±1318.7^3^ (2357–8402; 23)	5663.7±2070.7^1^ (2729–8970; 26)
f (kHz) of max power	7911.8±797.9 (5995–10120; 75)	4172.3±1166.4^3^ (2103–7044; 29)	5356.8±1740.1^3^ (2150–8600; 37)	5483.9±943.1^3^ (3389–7364; 46)	5831.7±1377.3^3^ (1620–8113; 81)	5589.6±1980.6^3^ (468–8501; 43)	3625.1±487.8^3^ (2962–4744; 18)	6919.1±1158.1^3^ (3604–8490; 22)	6267.9±1839.3^3^ (2923–9108; 28)
Max note bandwidth (kHz)	1033.7±828.3 (20–3622; 79)	2350.3±1686.8^3^ (188–6164; 55)	881.8±643.5^ns^ (165–3019; 37)	3028.8±1663.0^3^ (1004–6276; 56)	1768.8±1322.3^3^ (112–4811; 128)	1035.6±720.2^ns^ (255–3370; 53)	1260.5±513.5^ns^ (574–2198; 21)	1288.2±779.6^ns^ (176–2691; 24)	1050.5±1356.9^ns^ (68–6532; 28)
# of note repetitions	1.7±2.0 (1–12; 45)	1.2±0.6^ns^ (1–4; 25)	2.0±2.4^ns^ (1–11; 18)	2.4±2.6^2^ (1–11; 19)	2.3±2.2^1^ (1–10; 37)	3.4±2.6^2^ (1–7; 17)	1.6±0.9^ns^ (1–3; 15)	2.9±2.1 (1–7; 8)	3.2±3.4 (1–11; 9)
# of elements/motif	3.3±2.8 (1–13; 80)	3.8±2.3^1^ (1–10; 35)	5.4±3.3^3^ (1–15; 54)	3.6±2.6^ns^ (1–11; 25)	3.6±2.5^ns^ (1–10; 90)	6.3±4.9^2^ (1–17; 19)	2.9±2.0^ns^ (1–7; 12)	7.8±6.3 (1–20; 9)	4.9±3.4^2^ (1–13; 31)
# of motifs/strophe	6.6±3.6 (2–11; 8)	5.5±0.7 (5–6; 2)	14.0±4.2 (11–17; 2)	4.8±2.5 (1–7; 5)	4.7±2.6 (2–10; 19)	6.3±1.7 (4–8; 4)	5.3±0.6 (5–6; 3)	3.3±1.5 (2–5; 3)	5.5±3.0 (3–10; 6)
# of note types/strophe	8.3±4.0 (2–13; 9)	17.3±15.7 (5–35; 3)	17.3±3.7 (13–23; 6)	5.0±3.0 (2–9; 5)	6.6±2.7 (2–12; 18)	7.2±3.0 (3–15; 17)	7.0±1.4 (6–8; 2)	4.0±1.8 (2–6; 4)	7.3±5.1 (1–16; 15)
Note l (s)	0.1±0.1 (0.01–0.5; 75)	0.06±0.03^1^ (0.01–0.2; 27)	0.04±0.04^3^ (0.01–0.2; 37)	0.1±0.9^ns^ (0.01–0.3; 46)	0. 1±0.1^ns^ (0.02–0.4; 82)	0.04±0.04^3^ (0.004–0.2; 43)	0.1±0.04^ns^ (0.01–0.2; 21)	0.04±0.04^3^ (0.01–0.2; 23)	0.1±0.1^1^ (0.02–0.2; 26)
Internote interval (s)	0.05±0.1 (0.0–0.7; 59)	0.03±0.03^ns^ (0.0–0.2; 27)	0.03±0.03^ns^ (0.0–0.1; 29)	0.1±0.2^1^ (0.01–0.9; 34)	0.03±0.03^ns^ (0.0–0.1; 59)	0.02±0.02^ns^ (0.0–0.1; 35)	0.04±0.03^ns^ (0.01–0.1; 15)	0.05±0.06^ns^ (0.04–0.2; 21)	0.02±0.02^2^ (0.0–0.1; 23)
Intermotif interval (s)	0.3±0.4 (0.01–2.0; 68)	0.1±0.1^ns^ (0.03–0.4; 28)	0.2±0.1^3^ (0.01–0.6; 50)	0.3±0.3^ns^ (0.05–1.2; 17)	0.1±0.1^3^ (0.0–0.4; 72)	0.1±0.1^3^ (0,0–0.2; 17)	0.2±0.2^ns^ (0.01–0.5; 12)	0.6±0.4 (0.1–1.1; 7)	0.3±0.4^ns^ (0.02–2.2; 25)
Strophe l (s)	6.0±2.6 (2.4–10.8; 10)	5.0±6.1 (1.4–12.1; 3)	14.1±13.9 (4.3–35.8; 5)	2.9±1.8 (0.4–5.0; 6)	2.1±0.9^3^ (0.6–4.1; 18)	3.3±2.9^2^ (1.5–13.3; 17)	3.0±0.7 (2.3–3.7; 3)	12.0±20.6 (1.6–23.1; 5)	5.1±7.8^1^ 0.7–31.1; 14)
Interstrophe interval (s)	4.6±2.3 (2.2–9.0; 9)	3.0 (1)	3.3±2.1 (1.6–6.1; 5)	4.7±3.3 (1.9–9.5; 4)	5.7±2.8 (2.9–13.9; 16)	4.4±1.5 (1.6–7.6; 15)	8.0±3.2 (5.7–10.3; 2)	4.3±0.4 (4.0–4.6; 2)	5.2±3.3 (1.9–11.9; 9)
Clarity (0–10)	3.6±2.5 (1–9; 75)	4.9±1.9^3^ (2–8; 29)	4.0±1.6^1^ (2–7; 37)	4.1±2.3^ns^ (1–10; 46)	4.2±2.5^1^ (0–10; 75)	3.6±1.7^ns^ (1–9; 43)	7.0±1.7^3^ (3–9; 21)	5.7±2.7^2^ (2–9; 21)	4.8±2.1^1^ (2–8; 28)
Modulation (0–10)	4.3±3.0 (0–10; 75)	6.6±2.1^3^ (3–9; 28)	4.0±2.8^ns^ (0–9; 37)	7.5±2.9^3^ (0–10; 42)	5.3±3.4^ns^ (0–10; 76)	2.0±2.7^3^ (0–8; 43)	4.3±3.0^ns^ (0–8; 12)	4.0±2.8^ns^ (1–9; 12)	5.4±1.7^ns^ (2–8; 19)
# of harmonics	0.2±0.5 (0–3; 74)	0.4±0.8^ns^ (0–4; 28)	0.7±0.9^3^ (0–4; 37)	0.3±0.7^ns^ (0–4; 46)	0.2±0.4^ns^ (0–1; 83)	3.4±4.9^3^ (0–20; 43)	0.2±0.4^ns^ (0–1; 21)	1.1±1.3^3^ (0–5; 23)	0 (27)^1^
Harmonic strength (0–10)	2.5±1.2 (1–5; 14)	4.7±1.7 (1–6; 9)	2.4±0.9^ns^ (1–4; 18)	3.1±1.9 (1–6; 9)	3.7±2.8^ns^ (1–10; 14)	3.6±1.2^1^ (2–7; 19)	3.5±1.9 (1–5; 4)	4.7±2.2^2^ (1–7; 17)	–
Note similarity to next note (%)	61.2±31.0 (10–100; 65)	54.3±18.9^ns^ (2–9; 28)	61.3±24.9^ns^ (2–9; 32)	57.8±32.8^ns^ (10–100; 23)	66.5±26.9^ns^ (20–100; 71)	78.3±17.7^1^ (2–10; 40)	37.3±25.8^2^ (20–80; 15)	72.0±22.2^ns^ (20–90; 20)	75.2±21.7^ns^ (20–90; 23)
Strophe similarity to next strophe (%)	27.6±23.6 (0–60; 8)	75 (1)	77.5±5.0 (70–80; 4)	71.3±32.8 (30–100; 4)	27.0±34.1 (0–100; 15)	59.0±19.8 (20–90; 15)	80 (2)	50 (1)	50.0±54.8 (0–100; 6)
Note f trend (0–10)	5.3±2.0 (1–10; 63)	4.6±2.6^ns^ (1–9; 29)	4.6±1.9^ns^ (2–10; 37)	4.9±3.7^ns^ (0–10; 42)	5.7±2.5^ns^ (2–10; 79)	5.5±1.1^ns^ (3–8; 43)	4.5±1.4^ns^ (3–7; 13)	4.3±1.4^ns^ (2–7; 12)	4.9±1.7^ns^ (3–9; 20)
Motif f trend (0–10)	4.4±2.3 (0–10; 76)	5.7±2.1^2^ (2–10; 30)	4.8±2.1^ns^ (1–9; 50)	4.1±1.9^ns^ (1–7; 20)	3.9±1.7^ns^ (1–9; 76)	4.4±1.7^ns^ (1–7; 17)	4.6±0.9 (3–5; 5)	5.3±2.1 (2–8; 8)	4.3±1.3^ns^ (2–7; 28)
Relative max amplitude (0–10)	6.4±2.5 (0–10; 76)	6.4±2.2^ns^ (1–10; 28)	6.1±2.6^ns^ (1–10; 37)	8.2±1.7^3^ (1–10; 46)	6.9±2.3^ns^ (1–10; 83)	6.7±2.4^ns^ (2–10; 42)	6.3±2.0^ns^ (4–10; 21)	6.6±1.8^ns^ (4–10; 23)	7.1±2.2^ns^ (2–10; 28)

Mean ±SD (range, *n*); *n* listed under taxon name is number of individuals in recordings studied; *n* in each field is how many instances of the variable were measured or scored. Frequency (f) measures are in kHz, time measures are in s. P-values for significance tests (Mann-Whitney) between the new species and other taxa indicated as superscripts after SD for the other taxon: ^ns^ = p> 0.5;

1 = p≤0.05;

2 = p≤0.01;

3 = p≤0.001; no superscript indicates no test due to sample size <10.

Measurements confirm that all Asian *Muscicapa* species for which song is available (not including *M. striata* and *M. ruficauda*, which have very different songs) have similar song parameters, albeit based only on one to a few individuals of each species (Table 8). The song of the new species was significantly different from the congeners studied by between 5 (*M. s. sibirica*) and 9 parameters (*M. segregata*), with the other species having 7–9 statistically significant differences (Table 8).

**Figure 6:**
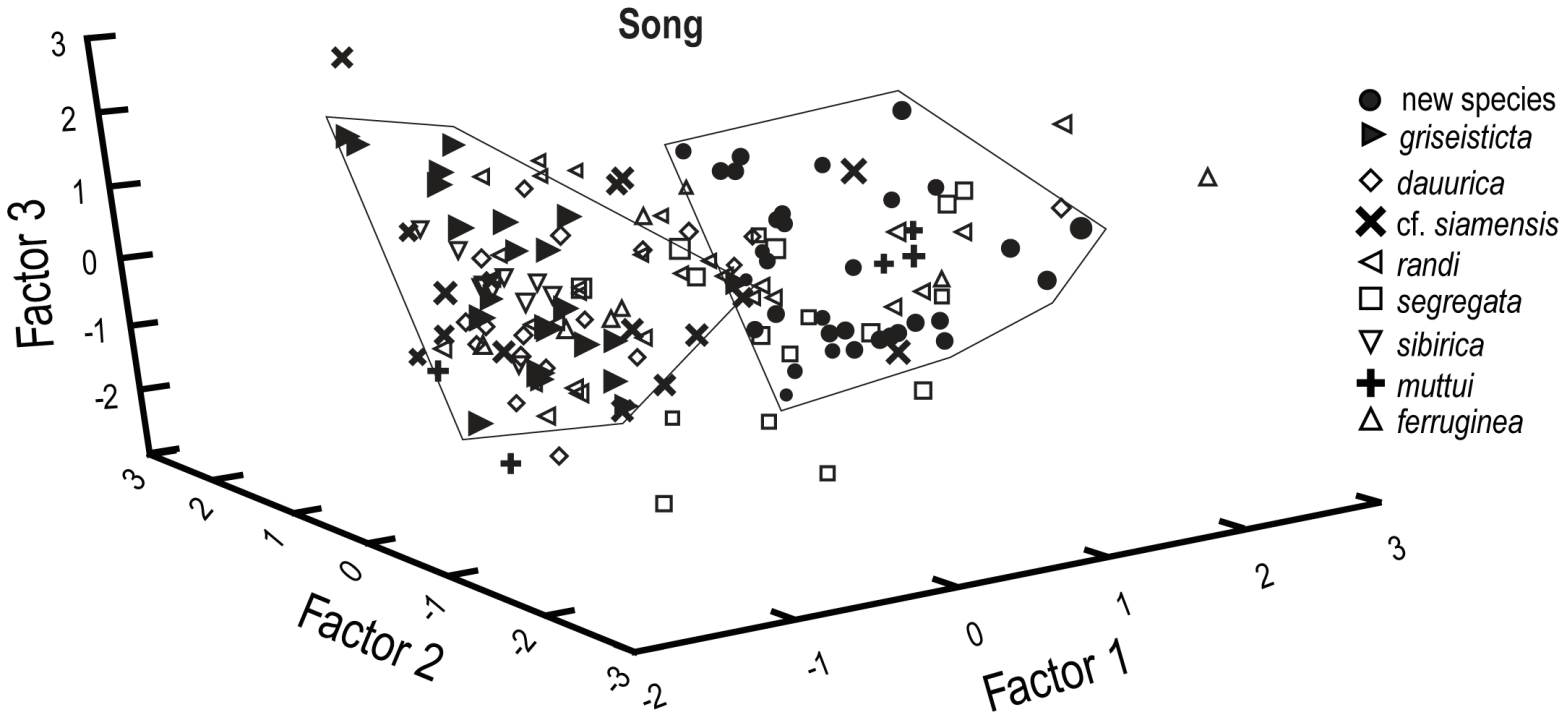
Principal components analysis of measurements of song characters for Asian *Muscicapa* taxa.

In a PCA of several song parameters, Factor 1 scores for individual song notes differ greatly between the new species and *M. griseisticta* (Figure 6; Table 9). There was more overlap, in some cases considerable, between song notes of the new species and most of the other taxa, although all the latter are more dissimilar in plumage from the new species than is *M. griseisticta*. The measurements that best separated the new species and *M. griseisticta* on PC-I were maximum note frequency, minimum note frequency, and frequency of maximum power, contrasted with several other measurements, note length, number of note repetitions, clarity, note bandwidth, number of harmonics, and note frequency trend (Table 9). Although PC 2 and PC 3 both explained an important amount of the variance, loadings on these axes did not separate the new species from the taxon most similar to it in plumage, *M. griseisticta*.

**Table 9:** Component loadings for principal components analysis of measurements of individual notes of Asian *Muscicapa* species.

Variable	Factor 1	Factor 2	Factor 3
Maximum note frequency	***0.91***	**0.33**	0.07
Minimum note frequency	***0.97***	−0.12	−0.02
Maximum note power frequency	***0.95***	0.13	0.04
Clarity	**−0.21**	***0.75***	0.14
Note length	0.10	***0.63***	***−0.47***
Note repetitions	**0.24**	***−0.61***	**0.39**
Note bandwidth	**−0.15**	**0.31**	***0.53***
No. of harmonics	−0.05	−0.11	***−0.44***
Note frequency trend	0.08	**−0.34**	***−0.54***
Eigenvalues	2.82	1.68	1.17
Percent total variance explained	31.33	18.66	13.04

Variables deemed most important in bold italic, those of somewhat lesser importance in bold only, and those not considered important in regular font.

### DNA

**Figure 7:**
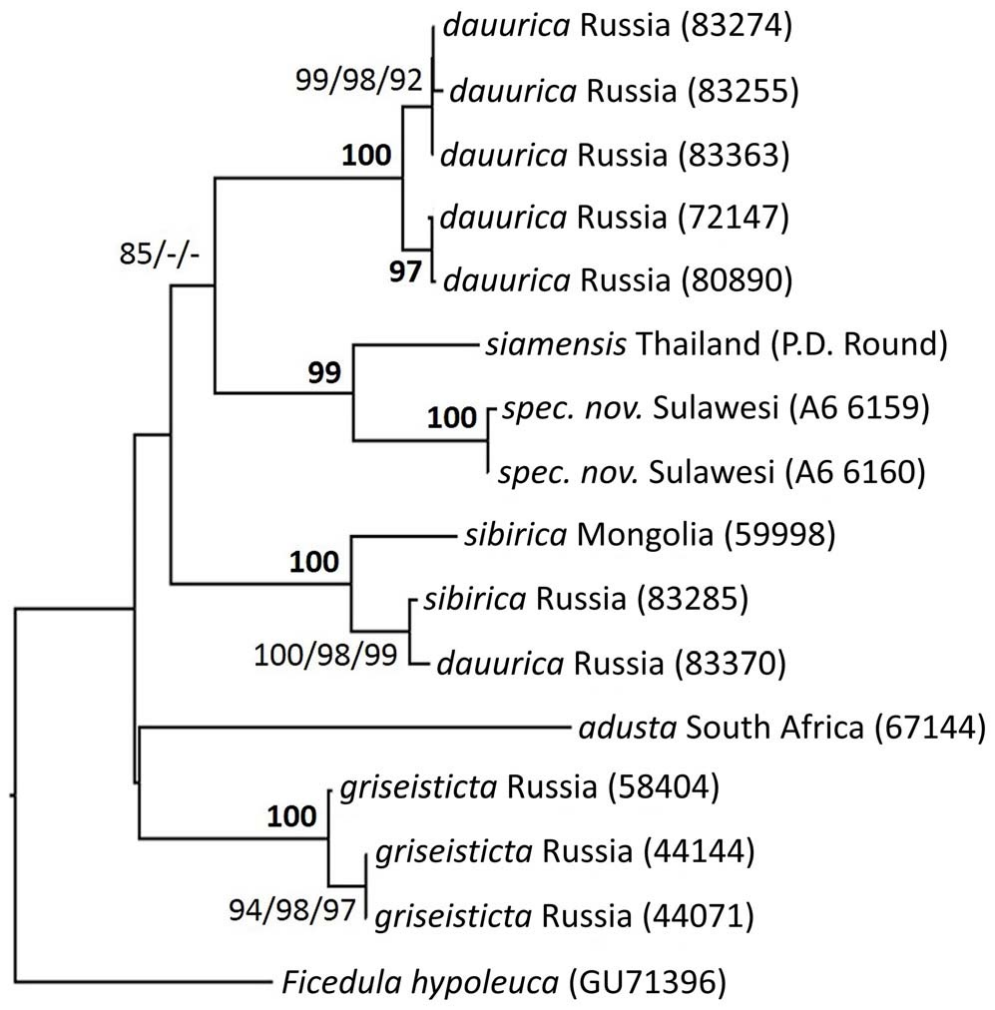
Neighbor-joining phylogram topology with bootstrap values for node support given for neighbor-joining, maximum-parsimony and maximum-likelihood analyses in the same order.

As all three phylogenetic analytical modes yielded identical topologies for strongly supported nodes, their results were easily combined into a single tree topology (Figure 7). The phylogenetic tree shows that the new species from Sulawesi is only distantly related to the superficially similar *M. griseisticta*, with which it co-occurs in the winter (Figure 7). Amongst Asian *Muscicapa* flycatchers, *M. griseisticta* is fairly diverged and does not emerge in positions of close affinity with any other species. *M. griseisticta* and the new species are diverged by 9.6–10.3% raw mtDNA sequence divergence, which is extremely deep for avian mitochondrial coding DNA divergences of members of the same species complex, and can often be found between members of different genera (see, e.g. ref. [24]).

Within our taxon sampling, the new species emerged closest to a sample of *M. dauurica siamensis* from Thailand, with a 4.1–4.3% raw mtDNA sequence divergence that is more typical of closely related species pairs although well above the level of mitochondrial coding sequence divergence usually encountered within avian biological species [24]. As the south-east Asian breeding *M. d. siamensis* did not emerge anywhere near nominate *M. d. dauurica* from Russia, our analysis confirms the necessity of on-going efforts to revisit currently recognized species limits within *M. dauurica*. However, for the purposes of this species description, the tree analysis suggests that both the taxon *siamensis* and the new species belongs to a clade of tropical and subtropical resident and short-distance migratory *Muscicapa* flycatchers that may have diversified within the south-east Asian region.

The tree analysis also confirms that plumage characters in *Muscicapa* flycatchers are a poor taxonomic indicator, as the new species turns out to be most closely related to a taxon to which it bears relatively little resemblance because of its extensively streaked breast and larger bill.

Based on our analyses of morphology, vocalizations, and mtDNA, there is no doubt that this is a new species, which we are honored to name:

***Muscicapa sodhii, sp. nov.***

Sulawesi Streaked Flycatcher

urn:lsid:zoobank.org:act: 54B169C6-CD09-455C-B80E-0FB178611BD4

### Holotype

**Figure 8:**
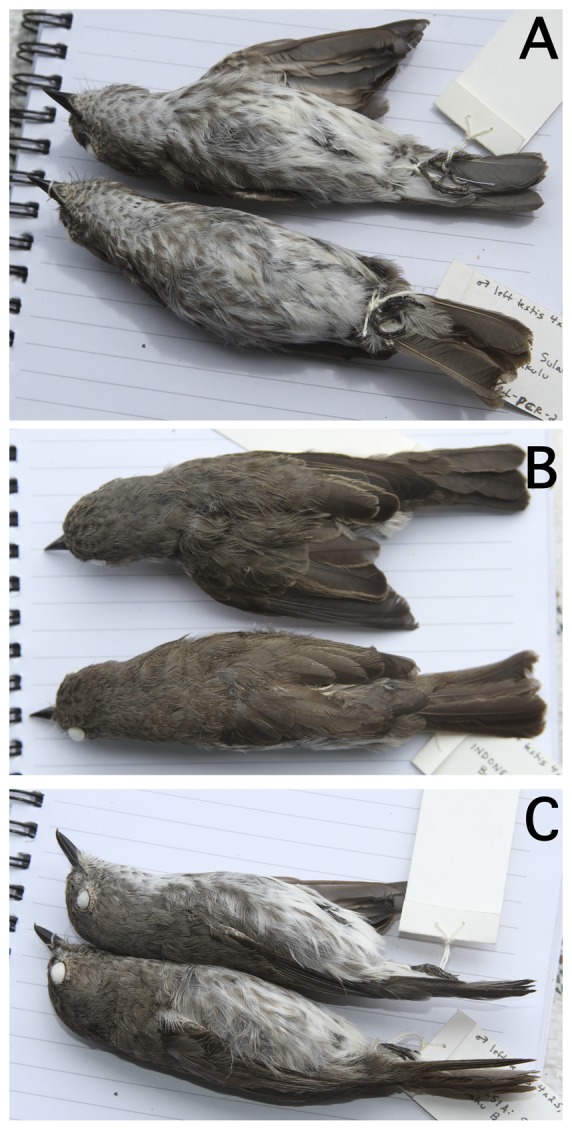
Photographs of the type specimens of the new species. (a) ventral view; (b) dorsal view; (c) lateral view. The paratype is above and the holotype is below in all photographs.

MZB.Ornit.33.525; adult male, collected 23 Jun 2012. Left testis 4×2.5 mm, pale tan in color; no brood patch; no cloacal protuberance (Plate 1, Figure 8). Skull 100% pneumatized; no bursa; no enlarged gape flanges. Little fat; stomach and intestine full of small black items (saved in alcohol). No head, body, wing, or tail molt. Collected by the Prawiradilaga field party; prepared by Pamela C. Rasmussen, field number LL-PCR-2012-010.

### Diagnosis

A small, drab gray-brown muscicapid flycatcher with indistinct facial patterning, strong dusky streaking below, and short primary projection. Differs from *Muscicapa griseisticta* in having the bill longer and more strongly hooked but relatively less broad; a weaker face pattern, with indistinct pale eyering (vs. prominent), dark spotting on throat (vs. mostly white throat), an ill-defined malar stripe and no pale moustachial stripe (vs. strong); much shorter and more rounded wing; shorter undertail coverts; and shorter, slightly more notched tail. Differs from *Muscicapa sibirica* in its longer, deeper, and, in Sino-Himalayan forms of *M. sibirica*, broader bill, much weaker head and throat pattern, much clearer streaking below, on a whitish background (vs. mostly dark background, especially in Sino-Himalayan forms); much shorter primary projection and first primary; shorter undertail coverts; and compared to *M. s. sibirica*, shorter and less notched tail. Differs from all forms of *Muscicapa dauurica*, as well as *M. segregata* and *M. randi*, especially in the strongly streaked underparts of *M. sodhii*. Differs additionally from *M. dauurica* in its shorter primary projection (longer only than *M. d. umbrosa*) and its more strongly hooked and, compared to *M. d. dauurica*, narrower bill; from *M. randi* and *M. segregata* in its shorter tail, less extensive pale area on lower mandible, and longer undertail coverts, and from *M. segregata* in its narrower bill and shorter tarsi.

The song of the new species is higher-pitched than that of all similar Asian species, and differs from them additionally in its combination of relatively narrow bandwidth, few note repetitions, mostly clear, longer notes, few harmonics, and low similarity between adjacent strophes.

### Description of holotype

Color terms using Munsell Color's [29] notation are shown in Table 10. Bill black, terete, with prominent culmen ridge and fairly strong hook, moderately broad, narrowing evenly toward tip, the distal fifth markedly narrow. Proximal third of lower mandible pale, distinctly so and sharply contrasting with black distal two-thirds of lower mandible, the pale extending around the ramal branches. Rictal bristles fairly prominent, moderately stiff, black. Forehead gray-brown with distinct dark brown centers and pale gray-brown edges, these markings larger on larger feathers of crown, and becoming obscure on feathers of rear crown. Nape and hindneck nearly uniform gray-brown. Sides of head uniform gray-brown, as nape. Lores mixed white and gray-brown, the feathers tipped dark giving a grizzled appearance, extends as narrow band over bill and as narrow (one feather row) indistinct eye ring, strongest in front of and below eye, weak above and behind eye. Pale lores grade into face below eye and sides of throat, which are indistinctly speckled. Chin and throat white, lightly and finely speckled gray-brown. There is no marked moustachial or malar streak; there is even gradation from pale grizzled lores to speckled throat and uniform gray-brown auriculars.

**Table 10:** Color comparisons of the holotype and paratype of *Muscicapa sodhii*, using Munsell (2000) soil color charts and notation.

Characteristic	MZB.Ornit.33.525, holotype, male	MZB.Ornit.33.530, paratype, unsexed
Color of crown feather edges	7.5 YR 5/1	7.5YR 6/1
Color of crown feather centers	7.5 YR 3/1	7.5 YR 2.5/1
Auriculars	7.5YR 4/1	7.5 YR 6/1
Neck sides	7.5YR 4/1	7.5YR 5/1
Mantle	7.5YR 4/1	7.5YR 4/1
Rump	7.5YR 4/1	7.5YR 4/1
Uppertail coverts	7.5YR 3/1	7.5YR 3/1
Throat streaks	7.5YR 6/1	7.5YR 5/1
Breast streaks	7.5YR 5/1	7.5YR 4/1
Flank streaks	7.5YR 6/1	7.5YR 4/1
Center of belly	pure white	pure white
Vent	pure white	pure white
Primaries	7.5YR 3/1	7.5YR 2.5/1
Internal edges of primaries	7.5 YR 8/2	7.5 YR 8/2
Tertial outer webs	7.5YR 5/2	7.5YR 4/1
Tertial inner webs	7.5YR 4/1	7.5YR 4/1
Tertial edges	worn off	7.5 YR 7/4
Primary underwing coverts	streaks 7.5YR 3/1	streaks 7.5YR 3/1
Axillaries	pure white	pure white
Uppertail feathers	7.5YR 3/1	7.5YR 2.5/1

Speckled throat grades into profusely streaked breast, which grades into more uniform brown breast sides and flanks, and less profusely streaked belly, especially center of belly. Flanks browner, less distinctly streaked than belly sides, and center of lower belly unmarked white. Ground color of entire underparts white. Vent unmarked white. Feathers of lower tibia white.

Mantle as nape. Feathers of back (below mantle) mostly lost during preparation due to shot damage, but same color as mantle. Rump and uppertail coverts plain gray-brown, as mantle. Primaries plain dark brown, darker than body plumage. Tertials paler, especially the outer webs. Secondaries as primaries but with very narrow pale edgings.

Marginal coverts (leading wing edge coverts; [30]) mixed dark brown and white. All upperwing coverts dark gray-brown with indistinct, broad pale grayer edges. Tail uniform drab dark brown, slightly paler and grayer on proximal outer feathers.

Soft part colors (from freshly dead specimen): Iris dark brown, narrow orbital ring black; bill black, basal third of lower mandible pale yellowish-cream; tarsi brownish-black, toepads grayish-tan, claws black.

### Measurements of holotype

Body weight 12.5 g; culmen from feathers 10.3 mm, wing length (flattened) 64 mm, tarsus 12.5 mm, tail 44.0 mm.

### Paratype

MZB.Ornit.33.530, unsexed adult (gonads destroyed by pellet), collected 25 Jun 2012 (Figure 8). No brood patch; no cloacal protuberance. Skull 100% pneumatized; no bursa; no enlarged gape flanges. Little fat; stomach full with several hard, rough, black, 2×2 mm seeds, beetle elytra, and other insect parts (contents saved in alcohol). Relatively large tapeworm found in pieces in abdominal cavity saved in alcohol. No head molt but body molt light, and molt present in primaries, secondaries, and rectrices. Collected by the Prawiradilaga field party; prepared by Pamela C. Rasmussen, field number LL-PCR-2012-014. Body weight 12.0 g; culmen from feathers 11 mm, wing length (flattened) 62 mm, tarsus 13.4 mm, tail 44.5 mm.

### Variation in the type series

The holotype is in worn plumage and was not molting, while the paratype was molting but mostly in fresh plumage. Differences in color and pattern are striking in direct comparison—the holotype is much browner above, the paratype much grayer. The holotype lacks distinct pale edges to tertials and secondaries, while the paratype has distinct buff edges to all these, narrow on secondaries and broader on tertials. The holotype has indistinct brown streaking from breast to flanks, with a white center of belly and vent; the throat brown-spotted, most densely on chin and least on lower central throat. The paratype has the same pattern below but the streaks below are bolder, grayer, and broader. The paratype (unlike the holotype) was prepared with one wing partially spread, and its primary underwing coverts are heavily dark streaked, looking mostly dark. The undersides of the primaries and secondaries are dark brown (paler in old feathers) with broad pale tan edgings, narrowest near tips. Both have similar face patterns, but while there is a narrow pale grizzled area over the bill that meets in the center in the holotype, this pale area is interrupted by the bill base in the paratype. The lower face (below plain auriculars) is blotchy, in the holotype with no discernible pale submoustachial and darker malar area, while in the paratype there is a moderately marked paler submoustachial and dark malar. Mantle feathers when fresh (as in the paratype) look uniform/monotone but actually have broad pale edges/tips on both sides then diffuse dark blotches. When worn (as in the holotype) the pale edges disappear and the feathers are then dark-tipped. Secondary greater coverts are vaguely paler tan-edged in fresh plumage (as in the paratype), not prominently as on secondaries and tertials. In worn plumage (as in the holotype), the wing feathers are much more uniform but the primaries look distinctly darker than the tertials, which wear to medium-pale brown, especially on the lateral webs.

### Specimens examined

No other specimens of the new species are known to exist.

### Etymology

The new species is named in honor of the late Professor Navjot S. Sodhi (1962–2011) for his monumental contributions to conservation biology and ornithology in Southeast Asia. Dr. Sodhi played a leading role in elucidating the effects of habitat disturbance on biodiversity, especially birds, across Southeast Asia (e.g. [31]–[36]). This research provided a fundamental understanding of the ecology of Southeast Asian forests and critical information required for conservation planning. In addition, Dr. Sodhi was an outstanding mentor for many students including D.L.Y. and J.B.C.H. for whom he was an honours and doctorate supervisor, respectively. The English name recognizes the endemic distribution of the species, with the descriptive term “Streaked” to avoid possible confusion with other endemic Sulawesi flycatchers.

## Remarks

### Distribution and habitat

**Figure 9:**
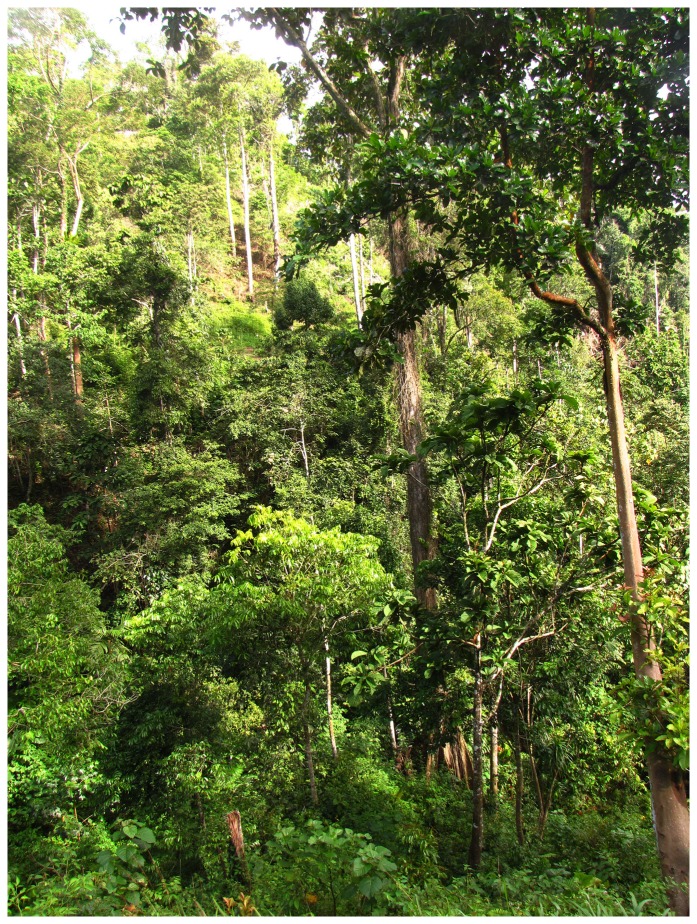
Habitat at the type locality. Mature cacao plantation with remnant tall forest trees at Baku Bakulu (photo by D.L.Y., 28 Jun 2012).

The species is known by our two specimens, the sightings and photographs of others, and our song recording from Baku Bakulu, Central Sulawesi. In 1997 King et al. [19] observed the flycatcher in a “very patchy remnant of forest” near Baku Bakulu. During our fieldwork in 2011 and 2012, the Baku Bakulu area was a mosaic of mature cacao plantations and small patches of remnant forest trees (Figure 9). This mixed agro-forest landscape abuts mature secondary and primary forest in Lore Lindu National Park. Despite the disturbance in the area, we found several forest-dependent species there, including Rufous-throated Flycatcher *Ficedula rufigula*, Sulawesi Dwarf Kingfisher *Ceyx fallax*, and Red-bellied Pitta *Pitta erythrogaster*. The flycatcher was seen at all levels in remnant forest trees in the agro-forest landscape.

There are also several sight records of *M. sodhii* from Danau Tambing, Badeaha, and the Sedoa area in the Napu Valley on the northeastern side of Lore Lindu National Park. So far, the species is not known from the western side of the park, where fewer birdwatchers visit, but some ornithological studies have been done [35],[37]. Other records in Sulawesi are based on photographs from Matabulu and Toraut, North Sulawesi and Karaenta, South Sulawesi. Despite the number of records to date, the distribution of *M. sodhii* remains poorly known. Most of the handful of sites where the species has been recorded, like Bantimurung-Burusaraung and Lore Lindu National Parks, are regularly visited by birdwatchers, and are where the species has been reported multiple times. However, the remote protected areas in Sulawesi's eastern and southeastern peninsulas are seldom visited and their avifaunas poorly known. The elevational range of *Muscicapa sodhii* is mainly within an elevational band of 150 m asl to 1,200 m in lowland and submontane evergreen forest and disturbed habitats. There are few records at higher elevations; the occasional observations of the species at Badeaha and Danau Tambing at c. 1,700 m may be exceptional and could involve post-breeding dispersal. While approximately half of the records are from primary lowland and submontane forests, the frequency of records (including that of the holotype and paratype) in disturbed habitats indicates that the species is tolerant of disturbance. It appears likely, however, that when it occurs in cacao plantations it requires the presence of at least scattered mature native forest trees.

### Vocal behavior

According to King et al. [19], the pair of birds they observed at Baku Bakulu gave high-pitched calls, which were recorded and played back, eliciting a noticeable response.

At 1702 h on 21 Jun 2012 at Baku Bakulu, J.B.C.H. found a singing individual of the undescribed flycatcher, sitting inconspicuously at mid-level near a fork of the main trunk of a large tree on a small hill-slope. The bird opened its bill noticeably while singing, and looked around a little, but otherwise sat quite still, making it hard to detect even though it was not hidden by foliage. After making a few recordings, P.C.R. played these quietly back to the flycatcher, which did not exhibit any obvious change in behavior but continued singing until 1707 h. It then flew off and we heard and recorded it singing again until 1722 h in a nearby densely vegetated area, but it did not respond noticeably to playback, nor was it seen again that day. Although differences in song before and after playback were not noted in the field, the later recordings (especially AV#17427–17428) made after playback are composed of short strophes, often of a single motif, while in the earlier-made cuts of the same individual most strophes are considerably longer and contain several motifs.

### Behavior

Although the behavior of *M. sodhii* remains poorly known, scattered documentation from local and visiting birdwatchers have allowed some aspects of foraging behavior and breeding ecology to be established. Like some other closely related *Muscicapa* flycatchers, *M. sodhii* appears to be an obligate insectivore that forages inconspicuously at all levels but perhaps mainly mid-levels. During our field work in 2011 and 2012, we observed single individuals sallying for flying insects, including large damselflies, from perches from several feet above ground to the subcanopy. Other known prey items as inferred from published photographs include orthopterans, including katydids (www.orientalbirdimages.org). Observations from other birdwatchers (e.g. [38]–[40]) indicate that the species may sporadically participate in mixed foraging flocks with other small passerines such as whistlers and white-eyes.

We did not observe breeding during our field work in May–Jun, but breeding details have been reported in three birdwatching trip reports. Farrow and Robson [41] and Hutchinson [42] observed adult birds attending single juveniles at Bantimurung-Bulusaraung in early Oct, while Hutchinson [43] reported one adult feeding a juvenile adjacent to Lore Lindu National Park near Wuasa in late Sep. The fact that breeding has only been reported in Sep–Oct despite multiple records (n = 17) from every month from Apr to Oct in the Lore Lindu and Bantimurung areas suggests that breeding, at least in central-south Sulawesi, may coincide with the start of the monsoon season. In North Sulawesi, the species is known to breed in May based on photographs deposited in the Oriental Birds Image database (www.orientalbirdimages.org) of a fledgling documented in May 2009.

### Diagnosability and species status criteria

Though superficially similar in appearance to *M. griseisticta*, *M. sodhii* is a well-marked species (see *Diagnosis*) that does not show close morphological approach to any other known taxon. In mtDNA it differs by at least 4.1% from all congeners, and it is highly distinct morphologically from the taxa that it is most similar to in mtDNA. Bearing in mind the small sample of singing individuals and that further vocal sampling is needed, *M. sodhii* also appears to differ in song from its congeners to a similar and in some cases to a stronger extent than the others do from each other. However, even if the available vocal sample proves unrepresentative, morphological and genetic differences clearly indicate species status. We therefore consider that, under any modern species concept, *M. sodhii* unambiguously fits the definition of a species and that its recognition as such will be non-controversial.

### Conservation

The fairly wide elevational and distributional range of *M. sodhii*, and its tolerance of habitat disturbance indicate that it is not immediately at high risk from logging or habitat conversion, which is ongoing in Sulawesi's lowlands and, increasingly, highlands [17],[18],[44],[45]. The species probably does not occur in young cacao monoculture, or where remnant forest trees are not preserved. It is a low-density species in the Lore Lindu National Park area and it appears to be uncommon elsewhere in Sulawesi, given that the species was only reported in 17 of 51 birdwatching reports we sampled since its first documentation in 1997. Nonetheless, the paucity of reports may be partly because the species had not yet been formally described. Present knowledge suggests the species does not approach the thresholds for ‘Vulnerable’ under the IUCN's range size or inferred population trend criteria [46]. We propose that the species be placed in the ‘Least Concern’ category.

Persistence of the species in small forest patches in a mosaic of cacao plantations over 15 years in Baku Bakulu shows that *M. sodhii* tolerates some level of disturbance and fragmentation. Our limited data however do not allow us to infer if disturbed habitats are preferred over primary forest. The scientific description of the species, including its voice, should allow comparative surveys to be done in forests and disturbed areas to learn about the species' habitat preferences. Furthermore, it should be a priority to survey poorly-sampled areas such as Sulawesi's eastern and southeastern peninsulas to collect distributional information on *M. sodhii*. Taxonomic studies are also needed to evaluate whether populations on Sulawesi's northern and southern peninsulas are distinct from Central Sulawesi birds. Many Sulawesi bird genera have racially or specifically distinct representatives that are allopatric on the island's peninsulas (e.g., *Heinrichia*, *Zosterops*, and *Ficedula*, among others), and it is possible that more than one taxon of *Muscicapa* is resident on the extraordinarily complex island of Sulawesi.
